# Genome-wide identification, characterization and expression analysis of the non-specific lipid transfer proteins in potato

**DOI:** 10.1186/s12864-019-5698-x

**Published:** 2019-05-14

**Authors:** Guojun Li, Menglu Hou, Yaxue Liu, Yue Pei, Minghui Ye, Yao Zhou, Chenxi Huang, Yaqi Zhao, Haoli Ma

**Affiliations:** 1 College of Agronomy, Northwest A&F University, Yangling, 712100 Shaanxi China; 2 Innovation Experimental College, Northwest A&F University, Yangling, 712100 Shaanxi China

**Keywords:** Potato, nsLTP, Genome-wide, Tuber development

## Abstract

**Background:**

Plant non-specific lipid transfer proteins (nsLTPs) are small, basic proteins that are abundant in higher plants. They have been reported to play an important role in various plant physiological processes, such as lipid transfer, signal transduction, and pathogen defense. To date, a comprehensive analysis of the potato *nsLTP* gene family is still lacking after the completion of potato (*Solanum tuberosum* L.) genome sequencing. A genome-wide characterization, classification and expression analysis of the *StnsLTP* gene family was performed in this study.

**Results:**

In this study, a total of 83 *nsLTP* genes were identified and categorized into eight types based on Boutrot’s method. Multiple characteristics of these genes, including phylogeny, gene structures, conserved motifs, protein domains, chromosome locations, and cis-elements in the promoter sequences, were analyzed. The chromosome distribution and the collinearity analyses suggested that the expansion of the *StnsLTP* gene family was greatly enhanced by the tandem duplications. Ka/Ks analysis showed that 47 pairs of duplicated genes tended to undergo purifying selection during evolution. Moreover, the expression of *StnsLTP* genes in various tissues was analyzed by using RNA-seq data and verified by quantitative real-time PCR, revealing that the *StnsLTP* genes were mainly expressed in younger tissues. These results indicated that *StnsLTPs* may played significant and functionally varied roles in the development of different tissues.

**Conclusion:**

In this study, we comprehensively analyzed *nsLTPs* in potato, providing valuable information to better understand the functions of *StnsLTPs* in different tissues and pathways, especially in response to abiotic stress.

**Electronic supplementary material:**

The online version of this article (10.1186/s12864-019-5698-x) contains supplementary material, which is available to authorized users.

## Background

Plant non-specific lipid transfer proteins (nsLTPs) were first discovered approximately 35 years ago from spinach leaves [1]. NsLTPs can transfer phospholipids from liposomes to the mitochondria or chloroplast [1, 2]. In general, plant nsLTPs are small, basic proteins with a size of approximately 6.5 to 10.5 kDa, which all have an eight cysteine motif (ECM) [3]. The common form of ECM is C-Xn-C-Xn-CC-Xn-CXC-Xn-C-Xn-C. These cysteine residues can be connected by four disulfide bonds to form a stable hydrophobic cavity structure [4, 5]. According to their molecular masses, the nsLTP proteins were initially divided into two types: nsLTP1 (9 kDa) and nsLTP2 (7 kDa) [1]. Later, depending on the sequence similarity of the ECM, nsLTPs were classified into nine types (Type I, II, III, IV, V, VI, VII, VIII, and IX) [4]. Then, two new types were defined: Type X [6] and Type XI [7]. The new classification method is not based on molecular size, but rather on the locations of conserved introns, the identity of amino acid sequences, and the spacing between Cys residues in the ECM [8]. This classification method is mainly applied to nsLTPs in lower plants [8].

NsLTPs, which account for approximately 4% of soluble protein, are abundant in higher plants [5]. NsLTPs are involved in many meaningful physiological and biochemical reactions in plant growth, including lipid transport between cell membrane systems, inhibiting the growth of pathogens, signaling and cell wall loosening [9, 10]. In vitro, plant nsLTPs are able to bind and transfer various types of hydrophobic molecules, including fatty acids, fatty acyl-CoA, phospholipids, glycolipids, and cutin monomers [10–12]. NsLTPs were found to accumulate massively in the apoplastic space and in the vascular system [13], which may be related to adaptation to different environmental stresses [14]. Plant epidermal cells specifically synthesize a waxy cuticle covering all surfaces of the plant to prevent water loss and pathogen intrusion. The cuticle consists of cutin polymers and soluble cuticular lipids [15–17]. To date, an increasing number of *nsLTPs’* functional studies have been reported in plants. For example, in *Arabidopsis*, *DIR1* encodes a non-specific lipid transfer protein that plays a vital role in plant resistance to the invasion of pathogens [18, 19]. In some cases, nsLTPs are thought to be associated with pathogenesis-related (PR) proteins. Therefore, it is considered to belong to the PR-14 family [20]. The *Arabidopsis* nsLTP-encoding gene *AZI1* is involved in signal transduction during bacterial infection [18, 21]. In *Arabidopsis*, *AtLTP2* plays a role in the integrity of the cuticle-cell wall interface and in etiolated hypocotyl permeability [22].

Potato is an important food and economic crop in the world. However, potato yield is susceptible to abiotic and biotic stress [23].In previous studies, overexpression of the *AtLTP3* gene enhanced performance of *Arabidopsis* seedlings under salt, drought, and cold stress [24, 25]. Overexpression of *GtLTP1* increases the resistance to *Botrytis cinerea*, indicating that this gene is a useful molecular tool for enhancing resistance of plants to pathogenic bacteria [26]. Studies have shown that *ltpg1* mutants in *Arabidopsis* are more susceptible to *Alternaria brassicicola* infection than wild type [27]. In addition, *StLTPa7* was shown to be involved in the early stages of resistance to *Ralstonia solanacearum* via a complex Ca^2+^-associated expression pathway in potato [28]. In addition, transgenic potato overexpressing *StnsLTP1* shows enhanced resistance to various abiotic stresses [29].

Plant non-specific lipid transfer proteins are widely distributed in the plant kingdom and are one of the well-known protein families. In total, 49, 52, and 156 *nsLTP* members have been identified in *Arabidopsis*, rice, and wheat, respectively [4]. In the *B. rapa* genome, there are 63, which can be grouped into nine types based on the diversity of the ECM [7]. In *L. japonicus*, 25 *nsLTPs* were identified and divided into seven types (I, II, III, IV, V, VIII, and IX) [30]. A database of 595 *nsLTPs* from 121 different species has now been established, with classification into five types (I, II, III, IV, V) [31]. Until now, only a small number of potato *nsLTPs* have been identified [6, 28, 29, 32]. With the completion of the whole-genome sequencing of potato [33], a genome-wide analysis of the *StnsLTP* genes can now be performed for the first time in our study. In this study, we identified 83 putative *StnsLTP* genes that can be grouped into eight types in the potato genome. A detailed analysis was carried out, including sequence analysis, phylogenetic relationships, gene structure, chromosomal location, gene duplication, GO annotation, and selective pressure analysis. Based on RNA-seq and the result of qRT-PCR, we performed the expression profiling of *StnsLTPs* in different tissues. The results obtained from this study laid the foundation for the further study of the function of *StnsLTPs* in potato, especially some genes that may have important functions in antibiotic processes and abiotic stress.

## Materials and methods

### Identification of *nsLTP* family members in potato

To identify candidate members of the *StnsLTP* family, several searches were conducted. First, the potato proteome and genome sequence were downloaded from the PGSC database (http://solanaceae.plantbiology.msu.edu/pgsc_download.shtml) [33]. Second, the published *Arabidopsis* and rice nsLTP sequences were retrieved and used as queries to identify the potato nsLTPs via the BLASTp and tBLASTn searches with a cutoff E-values of 1e^− 10^ and e^− 3^, respectively. In addition, we searched the PGSC database by name search using the name “nsLTPs” and “non-specific lipid transfer protein” as queries. To avoid missing potential StnsLTP members, we downloaded the HMM profile of plant lipid transfer proteins (PF00234) from the Pfam (http://pfam.xfam.org/) and used it as a query to for hmmsearch against the potato protein database by the HMMER 3.0 package [34] with the default parameters. After integrating the results of the above searches and eliminating redundancy, we also removed nsLTPs that did not contain an ECM domain. Additionally, within the candidate potato nsLTP proteins, a BLASTp search was performed on the protein sequences of 2S-albumin (At2S1 to At2S4) [35] and alpha-amylase inhibitor (RATI) [36] to exclude possible cereal storage proteins.

Finally, the potential nsLTP protein sequences were analyzed using the NCBI-CDD (https://www.ncbi.nlm.nih.gov/Structure/bwrpsb/bwrpsb.cgi) and Pfam websites. The sequences with incomplete domains and lack of nsLTP annotation were removed. The identified StnsLTPs were named in term of the method proposed by Boutrot et al. [4].

### Multiple sequence alignment and phylogenetic analysis

Multiple sequence alignment of the conserved ECM domains of the StnsLTPs was performed using ClustalX version 2.1 software with the default settings. Then, a phylogenetic tree was constructed by MEGA7 (http://www.megasoftware.net/) using the Neighbor-Joining (NJ) method with parameters as follows: Each node was calculated using 1000 repeated bootstrap tests [37], and the model/method was p-distance, and the gaps/missing data treatment was pairwise deletion.

### Identification of conserved motifs and gene structures in potato nsLTPs

To identify conserved motifs in the StnsLTPs, the MEME website (http://meme-suite.org/tools/meme) was used for identification with the default parameters except for the maximum number of motifs, which was set to 10.

The exon-intron structures of the *StnsLTP* genes were revealed using the GSDS2.0 website (http://gsds.cbi.pku.edu.cn/index.php). The genomic sequence and CDS sequence of the same *StnsLTP* gene were simultaneously submitted to the GSDS website to reveal its gene structure.

### Chromosomal locations and gene duplications of potato nsLTPs

Information about all the identified *StnsLTPs* was retrieved from the PGSC website. MapChart2.3 software (https://www.wur.nl/en/show/Mapchart.htm) was used to map the chromosomal positions and relative distances of the *StnsLTP* genes. In general, genes separated by no more than five other genes within 100 kb were considered as tandem duplications [38]. The segmentally duplicated *StnsLTP* genes were identified on the PGDD website (http://chibba.agtec.uga.edu/duplication/).

### Selective pressure analysis

The coding sequences for the duplicated genes were aligned by MEGA6 using the Muscle (codon) method. KaKs_Calculator2.0 Software [39] was used to calculate the Ka (the number of nonsynonymous substitutions per nonsynonymous site) and Ks (the number of synonymous substitutions per synonymous site) of duplicated genes by the MYN method. The divergence time “t = Ks/2r” was calculated with the neutral exchange rate r = 2.6 × 10^− 9^.

### Identification of cis-acting elements of potato nsLTPs

To investigate the cis-acting elements, the upstream regions of all the *StnsLTP* genes (1500 bp) were extracted from the Phytozome website (https://phytozome.jgi.doe.gov/pz/portal.html). Then, all of the sequences were submitted to the PlantCARE website (http://bioinformatics.psb.ugent.be/webtools/plantcare/html/) [40] to identify possible cis-acting elements.

### GO annotation and RNA-seq data analysis

To better comprehend the function of the *StnsLTP* genes, Blast2GO software (Version5.0) [41] was used to perform gene ontology (GO) analysis under the default parameters according to the following steps: (i) The full length amino acid sequences of the StnsLTPs were loaded into the Blast2GO software; [42] (ii) The BLASTp program built into the Blast2GO software was used to search the NCBI non-redundant protein database with the cutoff e-value(1e^− 10^); (iii) *StnsLTP* genes were mapped based on the BLASTp results; (iv) *StnsLTPs* were annotated. The annotation contained three categories: molecular function, cellular component, and biological process.

Processed RNA-seq data were downloaded from the PGSC database. The RNA-seq data of different developmental stages and different tissues of potato were selected to study the spatiotemporal specificity of *StnsLTP* gene expression. The expression patterns of *StnsLTPs* were analyzed in two varieties, DM (doubled monoploid) and RH (heterozygous diploid) [33]. The transcript abundance of the *StnsLTP* genes was represented by the FPKM (fragments per kilobase of exon per million fragments mapped) values. The heatmap of the *StnsLTP* genes was generated by the pheatmap package (https://CRAN.R-project.org/package=pheatmap). Genes with an FPKM value of zero in all tissues were excluded from the heat map.

### Plant materials

Potatoes (*S. tuberosum* cv. Atlantic), which were maintained in Dr. Chen’s laboratory at Northwest A & F University, were planted in a greenhouse at Northwest A & F University. During the growth of the potato, conventional cultivation techniques were used. Roots, leaves, stems, flowers, young tubers, and mature tubers were collected from potato plants at the young flowering stage to analyze the tissue-specific expression patterns of the *StnsLTP* genes. The different tuber developmental stages, including stolon (S1), tuber of the initial stage (S3), and mature tuber (S8), were defined as previously described [43]. In addition, there were at least three biological replicates and two technical replicates per tissue or organ. The collected samples were frozen without delay in liquid nitrogen and stockpiled at − 80 °C for later use.

### Quantitative RT-PCR analysis

Total RNA was extracted from the previously collected samples using the RNAsimple Total RNA Kit (BioTeke, Beijing, China). According to the manual of the RNA purification kit, the genome DNA contamination was digest in the RNA binding membrane with RNase free DNase I. The cDNA, which was synthesized by the PrimeScript reagent kit (Takara, Japan) in accordance with the manufacturer’s instructions, was diluted 5-fold for future analysis. Gene-specific primers for qRT-PCR were designed by Primer Premier 6 software (http://www.premierbiosoft.com/primerdesign/), which are listed in Additional file 1: Table S1. The qRT-PCR was performed on the Bio-Rad real-time system (CFX96, USA) using the KAPA SYBR FAST qPCR Kit Master Mix (2×) Universal (KAPA BIOSYSTEMS, Boston, United States). Three biological replicates and two technical replicates were analyzed. The qRT-PCR amplification system and amplification procedure were as previously described [44]. The internal reference gene was *ef1α*. The *StnsLTP* expression levels from different RNA samples were normalized as described previously [45]. The relative expression levels of the *StnsLTP* genes were calculated using a standard curve and normalized as described previously [46]. The results are shown as the mean ± standard deviation (SD).

## Results

### Genome-wide identification of StnsLTP genes

To identify all *StnsLTP* genes in potato, four bioinformatics methods were used in this study. In total, 77, 33, 54, and 128 *StnsLTP* genes were identified by keyword search, local BlastP search, local tBlastn search, and HMM search, respectively. After integrating the four parts of the results, sequences that contain incomplete ECM domains were manually deleted. Subsequently, 21 proteins translated by non-representative transcripts were removed. Next, a 2S albumin storage protein was also discarded. In addition, seven proteins that were annotated in the NCBI-CDD as incomplete proteins were also discarded. As a result, 83 sequences were identified as *StnsLTP* genes.

In previous studies, 28 *StnsLTP* genes were identified [6, 28, 29]. Then, 83 sequences were established based on the classification method (Table 1). However, several sequences could not be classified by the previous method. Hence, according to the similarity of the ECM domain sequences in amino acid sequences, those sequences were classified into two new types, XII and XIII. The genes were named according to their positions on the chromosome (Additional file 2: Table S2). The majority of the *StnsLTPs* had 0–2 introns, and only two genes (*StnsLTPIV.8*, *StnsLTPIV.10*) had 3 introns. The CDS sizes of the *StnsLTPs* ranged from 276 (*StnsLTPII.2*) to 666 bp (*StnsLTPIV.8*). Eight *StnsLTPs* (*StnsLTPI.6, StnsLTPI.19, StnsLTPI.24, StnsLTPI.28, StnsLTPVII.1, StnsLTPVIII.7, StnsLTPXII.1, StnsLTPXIII.6*) lacked an N-terminal signal peptide. The 83 identified StnsLTP proteins ranged in length from 92 (StnsLTPII.2) to 221 (StnsLTPIV.8) amino acids (aa) and had molecular weights (Mw) ranging from 9.77 kDa (StnsLTPII.1) to 22.88 kDa (StnsLTPIV.8). The theoretical isoelectric points (pI) were predicted from 4.16 (*StnsLTPXII.2, StnsLTPXII.4*) to 9.76 (*StnsLTPIV.3*).

**Table 1:** Some characteristics for the different types of non-specific lipid transfer proteins found in potato

Type	Number of members	Spacing pattern										
I	36	C	X_9–10_	C	X_12–17_	CC	X_19_	CXC	X_20–24_	C	X_3,13,14_	C
II	6	C	X_7_	C	X_13_	CC	X_8_	CXC	X_23,21_	C	X_6_	C
IV	10	C	X_9,10,18_	C	X_14–17_	CC	X_9,12,20_	CXC	X_24_	C	X_6–10,13_	C
V	2	C	X_14_	C	X_14_	CC	X_11_	CXC	X_24_	C	X_10_	C
VII	1	C	X_6_	C	X_14_	CC	X_12_	CXC	X_27_	C	X_8_	C
VIII	12	C	X_6,9_	C	X_12–14,16_	CC	X_12_	CXC	X_25_	C	X_8,9_	C
XII	7	C	X_9,10_	C	X_13,14_	CC	X_20,21_	CXC	X_21_	C	X_9,12,13_	C
XIII	9	C	X_8,9_	C	X_13–16_	CC	X_8,9,12,21_	CXC	X_19,21–23,26,30_	C	X_6–11,13_	C

The character “X” represents any kind of amino acid, and the Arabic numbers following “X” represent the number of amino acid residues

### Sequence analysis and classification of putative *StnsLTPs*

**Fig. 1:**
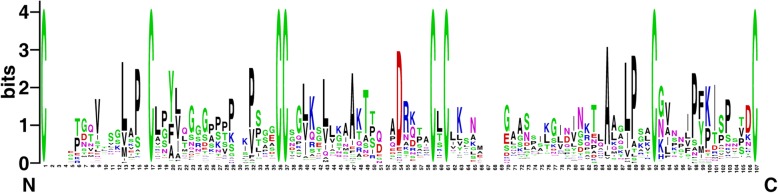
*StnsLTPs* were analyzed in a conservative domain through the WebLogo website. The height of the amino acid residue at each position represents the degree of conservation of this amino acid residue. The number on the x-axis indicates its sequence position in the corresponding conserved domain. The y-axis represents information measured in bits

Because the potato is a flowering plant [8], we used the sequence similarity method to classify *StnsLTPs*. Based on the classification scheme of Boutrot et al. (2008) and Liu et al. (2010), we added two new types of *nsLTP* (types XII and XIII) based on the sequence similarity of the ECM [4, 6]. The 83 *StnsLTPs* were divided into 8 types (I, II, IV, V, VII, VIII, XII, and XIII); none were classified as type III, VI, X, or XI (Table 1 and Additional file 2: Table S2). Interestingly, type II potato *nsLTPs* were single-exon genes. This result was consistent with other studies in *B. rapa*, *Gossypium spp.* and *Solanum lycopersicum* [12, 32] . As shown in Table 1, the difference between the new type XII and XIII was mainly the number of amino acids between the fourth cysteine residue and the fifth cysteine residue. The eight Cys residues in all 83 *StnsLTPs* were found to be highly conserved in structure (Fig. 1 and Additional file 5: Figure S1). Of the 83 *StnsLTP* genes we identified, the members of type I accounted for the largest proportion (43.37%).

### Chromosomal locations and gene duplications of StnsLTP genes

**Fig. 2:**
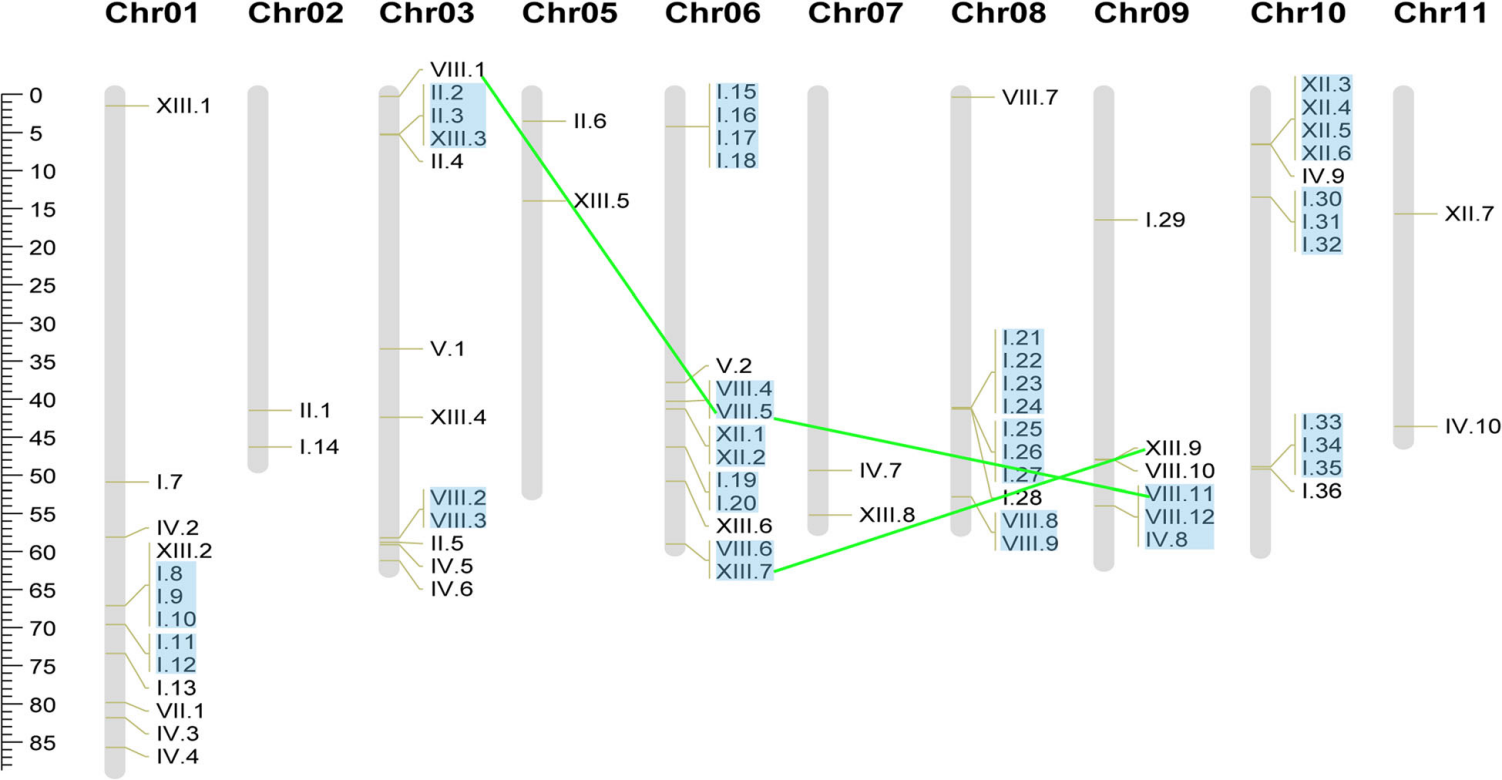
Chromosomal localization and gene duplication of *StnsLTPs.* The tandem duplicated genes are marked with blue rectangles, and the segmentally duplicated genes are linked by green lines. The positions of the *StnsLTP* genes on the chromosome can be inferred from the ruler on the left, and the units of the ruler is megabases (Mb)

To demonstrate the exact locations and distributions of the *StnsLTP* genes on different chromosomes, we constructed a detailed chromosome map. The positions of the *StnsLTP* genes were downloaded from the PGSC database. Mapchart software version 2.3 was used to generate the chromosome map. Seven genes, including *StnsLTPI.1*, *StnsLTPI.2*, *StnsLTPI.3*, *StnsLTPI.4*, *StnsLTPI.5*, *StnsLTPI.6*, and *StnsLTPIV.3*, could not be mapped to any of the potato chromosomes. The rest of the *StnsLTPs* were randomly distributed on ten chromosomes, with none on chromosomes 04 and 12. The largest number of *StnsLTP* genes were found on chromosome 06 (14 genes). In addition, 13 *StnsLTP* genes were located on chromosome 01; 11 members on chromosomes 03 and 08; two members on chromosomes 02, 05, 07, and 11; five on chromosome 09; 12 on chromosome 10 (Fig. 2).

In the process of evolution, both tandem duplication and segmental duplication are a pivotal part in the generation of gene families [47]. Therefore, *StnsLTP* gene duplication events were analyzed. According to the general criteria (two genes are separated by fewer than five other genes within a 100 kb chromosome fragment [38]), 16 gene clusters were regarded as tandem-duplicated genes, accounting for 53.01% (44 out of 83). According to the data downloaded from the PGDD website, three pairs of *StnsLTP* genes were identified as segmentally duplicated genes (Fig. 2).

In addition, the Ka/Ks value for each pair of duplicated genes was calculated to estimate the rate of molecular evolution (Additional file 3: Table S3). The value of Ka/Ks is conventionally used as a sign of selection pressure [48]. Ka/Ks > 1 indicates a positive selection effect, while Ka/Ks < 1 indicates that a purifying selection exists between the duplicated genes; Ka/Ks = 1 suggests that a neutral selection exists between the duplicated genes [49]. Our results showed that 91.49% of the duplicated *StnsLTPs* underwent purifying selection pressure during the duplication events (Additional file 3: Table S3), which showed that the function of duplicated *StnsLTPs* might not have changed much in the subsequent evolutionary process. In addition, the divergence time between duplicated gene pairs was also calculated using the previously described formula (Additional file 3: Table S3). The Ks values of the vast majority of *StnsLTPs* were greater than 0.52 and the corresponding divergence time might be greater than 100 million years ago (MYA). Interestingly, the Ks value of one tandem duplicated gene pair (*StnsLTPI.24/StnsLTPI.21*) was 4.92384, and the corresponding duplication age might be 946.89 million years ago (MYA) (Additional file 3: Table S3).

### Phylogenetic analysis of potato, rice and *Arabidopsis* nsLTPs

**Fig. 3:**
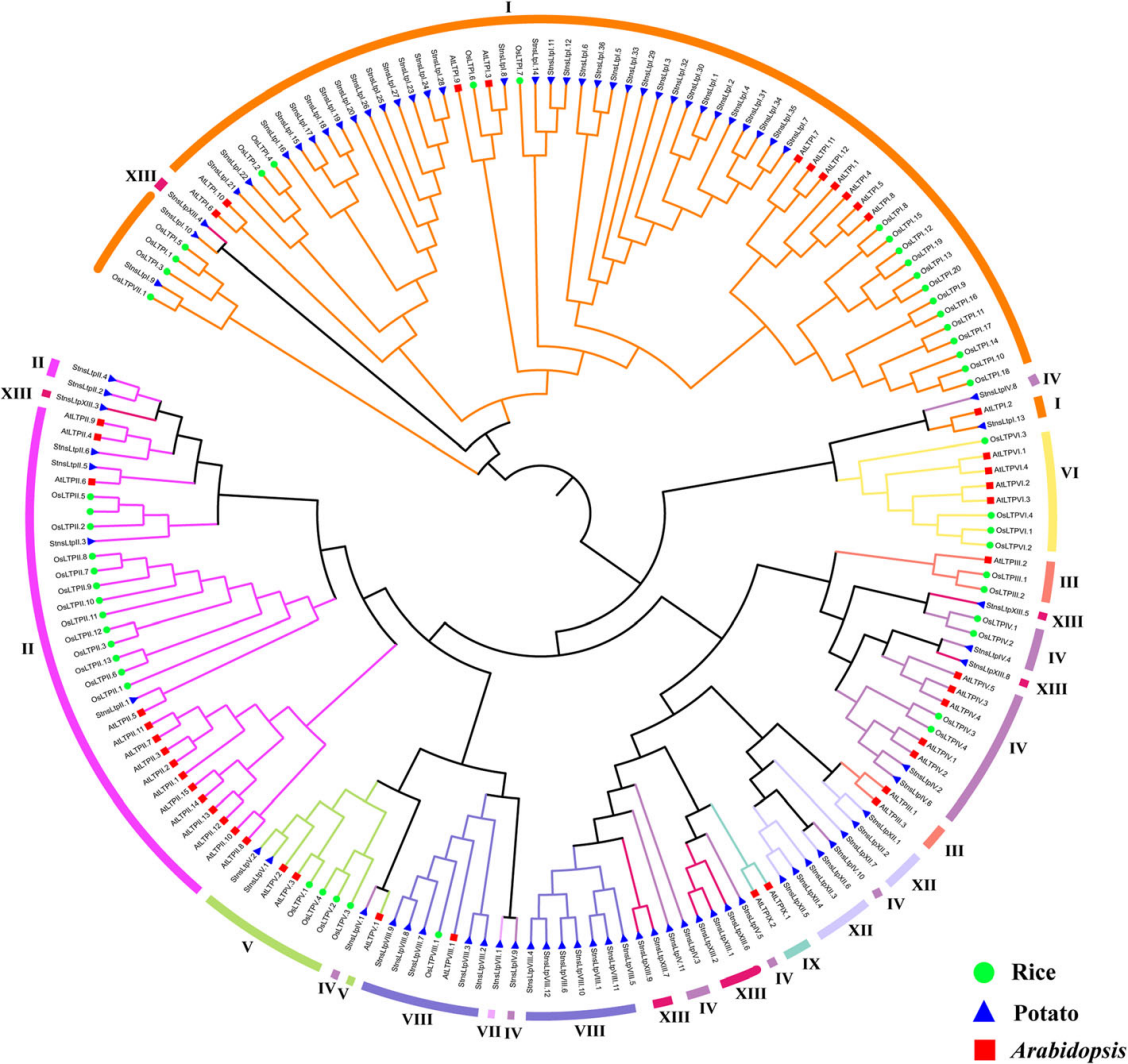
A phylogenetic tree of *Arabidopsis*, rice, and potato nsLTP proteins**.** A phylogenetic tree was constructed using the NJ method using MEGA7 software, and 1000 bootstrap tests were performed. Different types are marked with different colors

To better understand the evolutionary relationship among nsLTPs, a phylogenetic analysis of nsLTPs identified in potato, *Arabidopsis*, and rice was performed using ClustalX2.1 and MEGA7 software. In total, 177 nsLTPs from the three species were analyzed. We performed multiple sequence alignments with previously identified ECM conserved domain sequences of potato, rice, and *Arabidopsis*. A phylogenetic tree was constructed by the NJ method as described above (Fig. 3). It was noteworthy that nsLTPs were more closely related to members of the same type than other types of nsLTPs from the same species, suggesting a relatively high degree of homology within the same type of nsLTPs from different species. Among the eight types of StnsLTPs, only the distributions of only type XIII and IV were relatively random. Therefore, the two new types of StnsLTPs may have evolved from other types of *StnsLTPs*.

### Conserved protein motifs and gene structure of potato *nsLTPs*

**Fig. 4:**
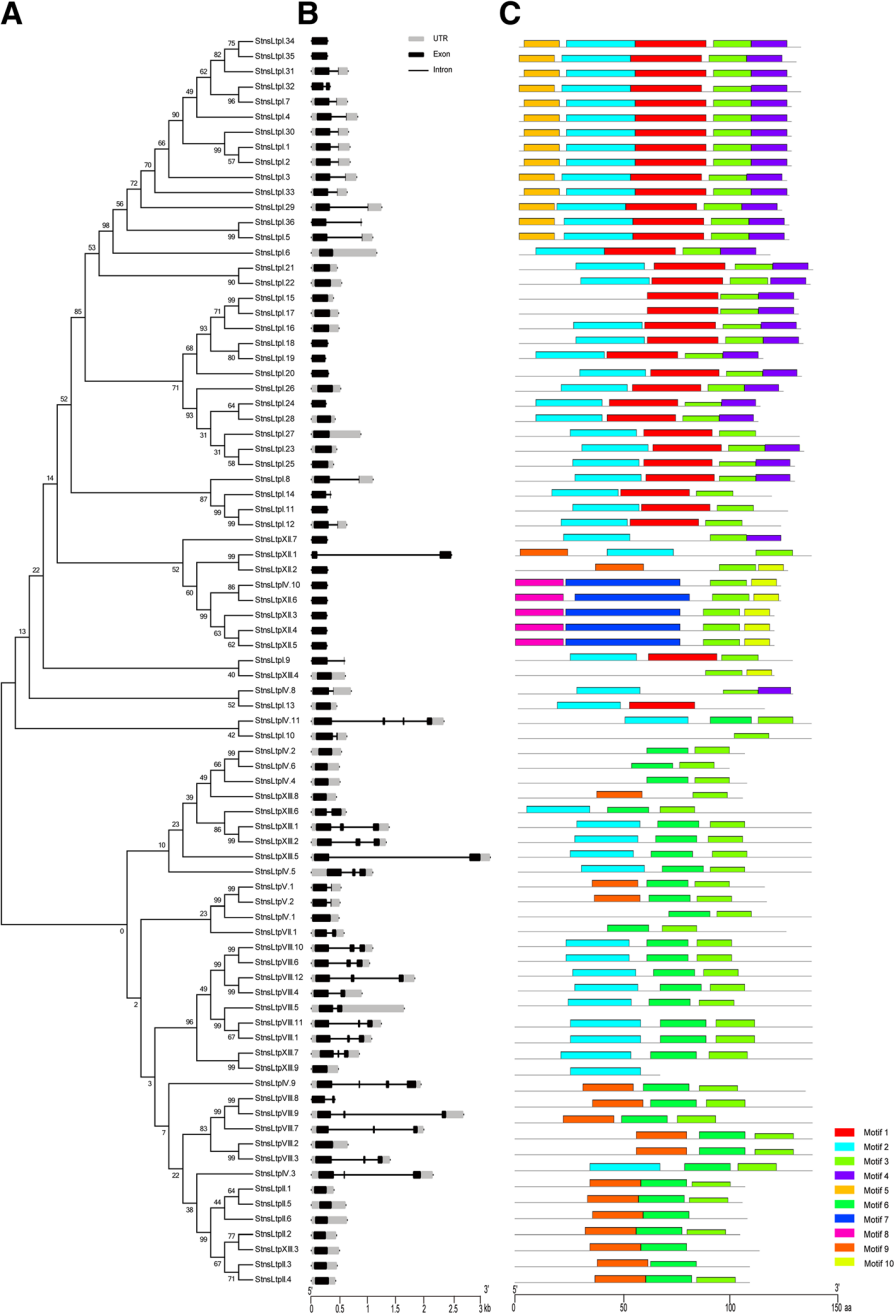
Phylogenetic relationships and gene structure of the *StnsLTP* genes and conserved motifs of the StnsLTP proteins. A, Phylogenetic tree of 83 StnsLTP proteins. A rootless Neighbor-Joining phylogenetic tree was constructed using MEGA7 software with the full-length amino acid sequences of 83 StnsLTP proteins, and the bootstrap value was set to 1000. B, A conserved motif distribution map in the *StnsLTP* gene. The 10 predicted motifs are represented by different colored boxes. C, Exon/intron maps of *StnsLTP* genes. Black boxes represent exons, and black lines represent introns. The UTRs region of the *StnsLTP* genes are represented by a gray box. The lengths of the introns and exons can be inferred by the ruler at the bottom

The major feature of plant nsLTPs is the highly conserved ECM. Using the ECM sequences of the nsLTPs identified in potato, sequence logos were visualized by the WebLogo website (http://weblogo.berkeley.edu/logo.cgi) (Fig. 1). Then, to better understand motif conservation in *StnsLTPs*, ten motifs were identified by the MEME website (Fig. 4c). In addition, the results of the multiple sequence alignment showed a change in the number of amino acid residues between the eight conserved cysteines, which contributed to the classification of different types of *StnsLTPs* (Additional file 5: Figure S1).

Gene organization takes part in the evolution of multigene families [50]. Therefore, the intron-exon structure of the 83 *StnsLTP* genes were analyzed. As shown in Fig. 4b, type I, which included more than half (52.79%) of the *StnsLTP* genes, didn't have intron, and the remaining member of *StnsLTPs* had one intron. Interestingly, members of type II didn't contain intron, members of type V and VII had only one intron, and type IV members had a wide variety of intron structures. There was one intron in *StnsLTPIV.8*, two introns in *StnsLTPIV.4* and *StnsLTPIV.6*, and three introns in *StnsLTPIV.7*, *StnsLTPIV.9*, and *StnsLTPIV.11*. The remaining five members didn't have intron in their gene structure. In type XII, only *StnsLTPXII.1* had one intron, and the remaining members didn't have intron. In type XIII, there was one intron in *StnsLTPXIII.5* and *StnsLTPXIII.6*, two introns in *StnsLTPXIII.1*, *StnsLTPXIII.2* and *StnsLTPXIII.7*, and the remaining genes didn't have intron. In summary, members of the *StnsLTP* gene family contained small numbers of introns (maximum of three) or didn't have intron.

### Gene ontology analysis of potato nsLTPs

**Fig. 5:**
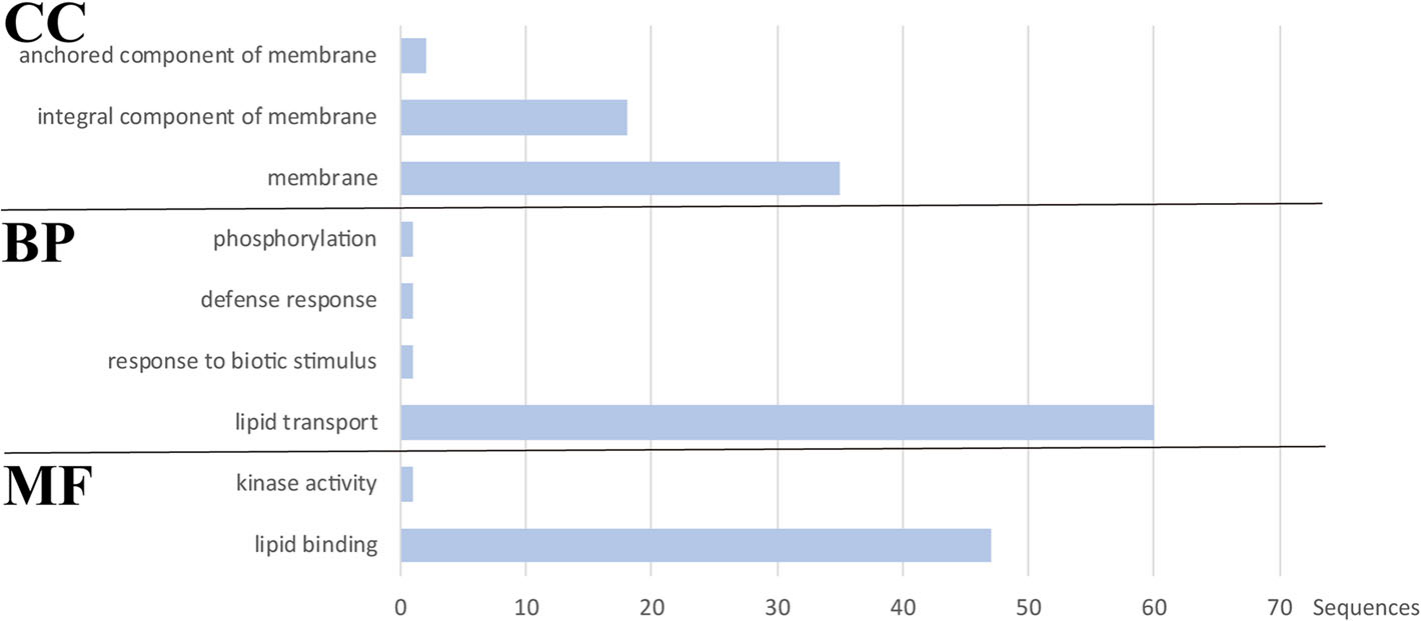
Gene Ontology (GO) annotation of the StnsLTP protein. The result of the annotation includes three parts: MF, molecular function. BP, biological process. CC, cell composition

To better understand the molecular functions, biological processes and cellular components of StnsLTPs, gene ontology (GO) analysis was performed. As shown in Fig. 5 and Additional file 4: Table S4, only 48 StnsLTP proteins were annotated in terms of molecular function. In total, 47 StnsLTPs were annotated as lipid binding. Surprisingly, StnsLTPIV.9 was annotated as kinase activity. Only 62 StnsLTPs were annotated in the category of biological process. The annotated StnsLTPs were involved in a variety of biological processes. Most StnsLTPs (96.77%) took part in lipid transport. In addition, StnsLTPI.29 could participate in multiple biological processes, such as defense response and response to biological stimuli. It is noteworthy that the involvement of StnsLTPIV.9 in the phosphorylation process was related to its molecular function. Based on the biological process analysis, the main functions of StnsLTPs were to bind lipids and participate in some other biological processes such as abiotic and biotic stress responses and signal transduction. In addition, 55 StnsLTPs were annotated in a cellular component. The results showed that StnsLTPs were located on the cell membrane, which was related to their function of transporting lipids.

### Key cis-acting elements within the promoter regions of *StnsLTPs*

Cis-acting elements that located in the promoter are the binding sites for transcription factors, and thus, they are also essential for regulating the initiation of gene transcription [40]. To further explore the regulatory mechanisms of *StnsLTPs* in development and abiotic and biotic stress response, a 1500 bp upstream sequence from each transcription start site was submitted to the PlantCARE website [40] for identification of cis-acting elements. Because potato is an endosperm-free species, we did not show cis-acting elements associated with endosperm development in the results. As shown in Additional file 6: Figure S2, 18 types of cis-acting elements were identified. The *StnsLTPs* contained a large number of stress-responsive cis-acting elements. All cis-acting elements could be divided into three types according to their different functions. Type A cis-acting elements were all related to plant hormones, including P-box, CGTCA-motif, TGACG-motif, ERE, TGA-element, and TATC-box. Type B cis-acting elements were related to plant response to biotic and abiotic stress, including Box-W1, WUN-motif, TC-rich repeats, LTR, MBS, HSE, and TCA-element. Type C cis-acting elements were related to regulating plant growth and development, including ARE, CCGTCC-box, CAT-box, circadian, and 5UTR Py-rich stretch.

### Expression patterns of *StnsLTP* genes

**Fig. 6:**
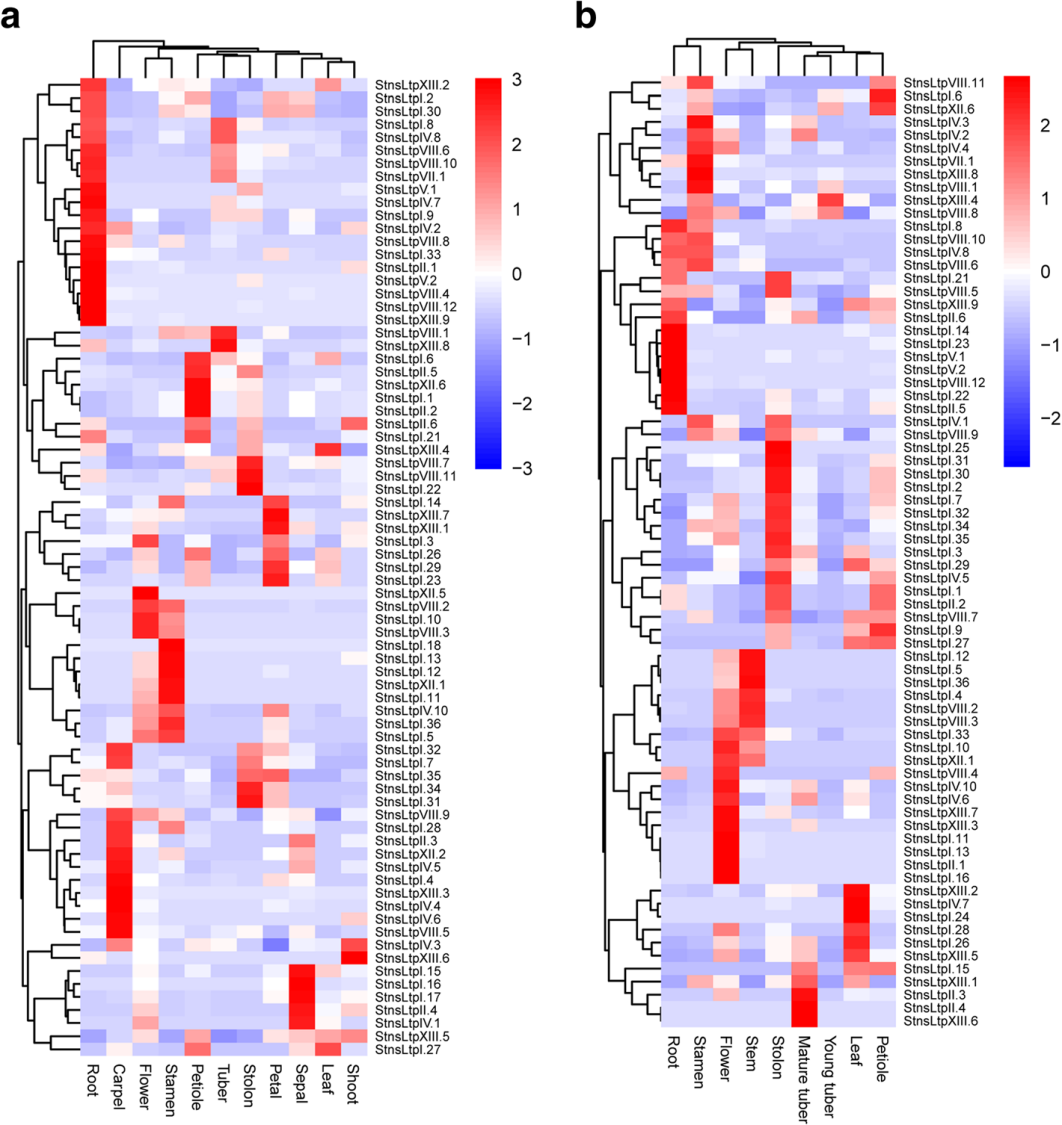
Expression profiles of *StnsLTP* genes in double monoploid (**a**) and heterozygous diploid (**b**). The FPKM value of the *StnsLTP* gene was generated from the heatmap of the pheatmap package in the R software

To better elucidate the expression patterns of *StnsLTPs*, we analyzed the public RNA-seq (RNA sequencing) data available on the PGSC website. A heatmap was generated except for *StnsLTPI.20*, for which the FPKM value could not be found. As shown in Fig. 6a and b, the expression of the *StnsLTPs* showed temporal and spatial specificity and with a slight difference between varieties, which may be due to different genetic backgrounds. Interestingly, most of the *StnsLTP* genes were expressed in flower organs in both DM and RH. Most of the *StnsLTP* genes were highly expressed only in a specific organ or at a specific developmental stage. For example, the *StnsLTPV.1*, *StnsLTPXIII.9*, *StnsLTPV.2*, and *StnsLTPVIII.12* genes have high expression in the root but low expression in other organs. Similar to previous studies, the *StnsLTPVIII.1*, *StnsLTPXIII.4*, and *StnsLTPVIII.8* genes were found in young potato tubers and were specifically expressed at low levels in mature potato tubers.

### Expression analysis of *StnsLTPs* by quantitative RT-PCR

**Fig. 7:**
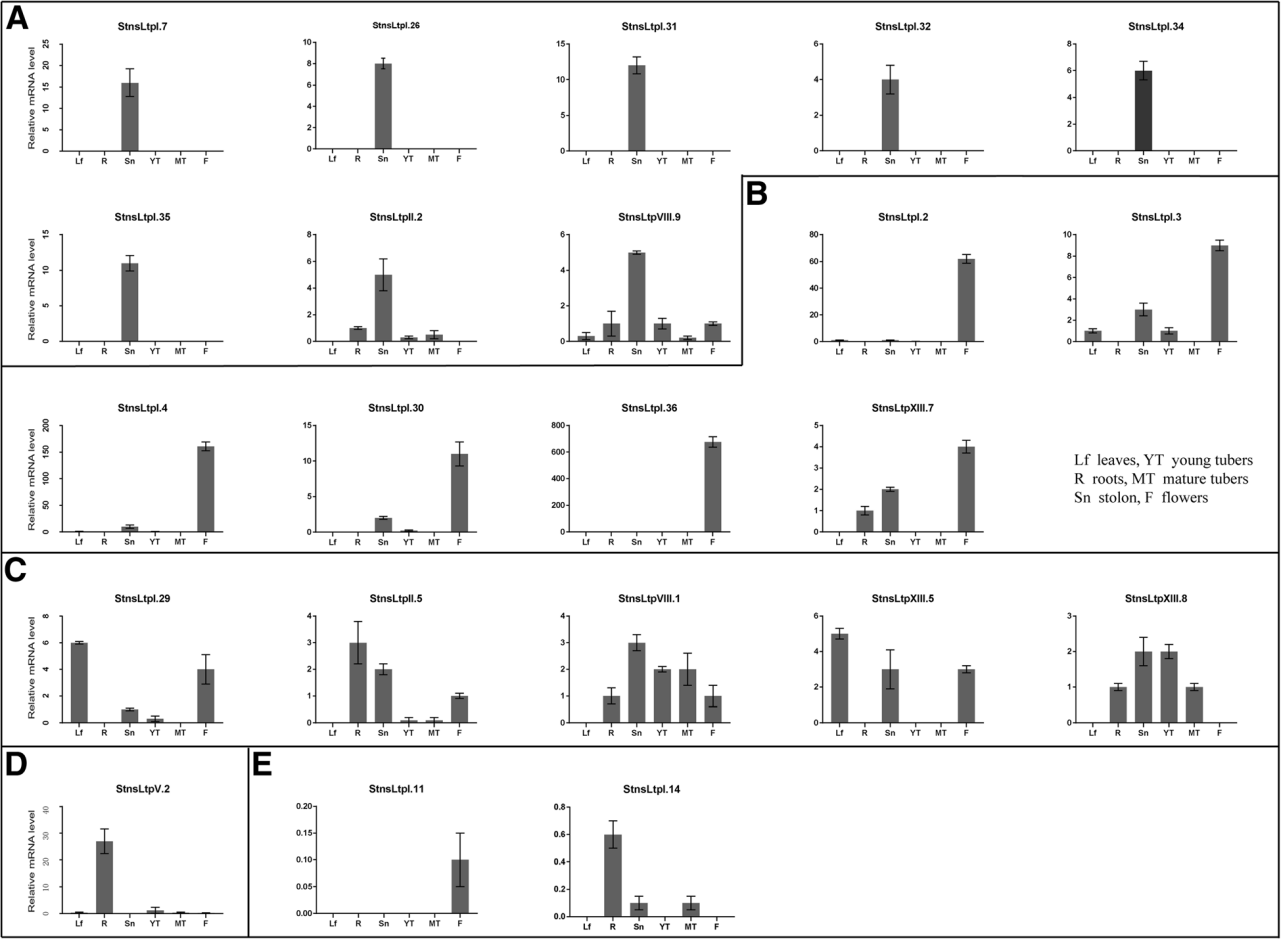
Expression profiles of *StnsLTP* genes in six tissues. Quantitative RT-PCR was used to study the expression levels of 22 selected *StnsLTP* genes. The results were expressed as the mean ± standard deviation. The internal reference gene used in qRT-PCR was *ef1α*

To better understand the biological roles of the *StnsLTP* genes, 22 *StnsLTP* genes were selected based on RNA-seq data and gene-specific primers were designed. Six tissues, including young leaf (Lf), root (R), stolon (Sn), young tuber (YT), mature tuber (MT), and flower (F) of potato cultivar Atlantic, were analyzed by quantitative RT-PCR. As shown in Fig. 7, on the basis of their expression patterns, these 22 *StnsLTP* genes were divided into five groups (A-E). Genes of group A were specifically expressed in the stolon. Among them, *StnsLTPI.7*, *StnsLTPI.26*, *StnsLTPI.31*, *StnsLTPI.32*, *StnsLTPI.24.* and *StnsLTPI.35* were mainly expressed in the stolon, while the other two genes (*StnsLTPII.2* and *StnsLTPVIII.9*) were also expressed in other organs. The genes of group B were specifically expressed in the flower. Compared with the other analyzed genes, the C group of genes showed diverse expression patterns with expression in multiple tissues. For example, *StnsLTPI.29* had the highest expression level in the leaf, but it also showed some expression in the flower and other tissues. *StnsLTPV.2*, which was the only gene in group D, was mainly expressed in the root. Both genes of the E group were barely expressed in all the sampled organs. The expression levels of *StnsLTPI.11* and *StnsLTPI.14* in the six organs were very low, suggesting that they might have a special spatiotemporal expression pattern, but they were not detected in the qRT-PCR data.

## Discussion

Currently, potatoes are an important part of the world’s food supply. However, during growth and development, potatoes are subjected to various stresses [51, 52]. Plant nsLTPs, which are abundant in higher plants, account for approximately 4% of soluble protein and play a unique role in plant life activities [22, 29, 53, 54]. This gene family has been identified in *Arabidopsis*, rice, wheat, maize, *B. rapa* and other plants [4, 7, 10, 32, 42]. However, little is known about the *nsLTP* gene family in potato, because the potato genome-wide sequence has only recently been published [33].

In this study, we identified 83 potential potato *nsLTP* genes in silico, which were classified into eight types (I, II, IV, V, VII, VIII, XII, and XIII), of which the XII and XIII types are new types identified herein; no Type III, VI, IX and XI *nsLTPs* were found in potato. A previous study of six *Solanaceae* species showed that four types of *nsLTP* (III/V/VI/VIII) were not identified [6]. Our results are similar to the previous study. However, there are 12 *nsLTPs* of type VIII in this study. Why do Type III/V/VI *nsLTPs* have so few members, while the number of type VIII *nsLTPs* suddenly increased in the potato genome? This is still a question that needs further study. It is worth noting that we identified a gene belonging to type VII, which contains 27 amino acid residues between Cys6 and Cys7, although it is speculated that type VII *nsLTPs* occur only in monocots [4, 6]. Therefore, we believe that the first reason is the lack of precise mapping of genome sequencing, and the second reason is due to the complexity of the potato genome.

Although many potential *nsLTPs* have been identified based on the whole-genome sequences in the plant kingdom, only a small number of *nsLTP* functions have thus far been elucidated. As the research progresses, increasing evidence has shown that *nsLTPs* are likely involved in many biological processes, such as signal transduction, reproduction, and response to biotic and abiotic stresses [3]. Unfortunately, the potato is often subjected to abiotic stress, which in turn leads to poor plant growth and ultimately yield loss. For example, when the potato is exposed to water stress during the development period, its yield will decrease as the stress level deepens [55].

As an evolutionary relic, intron-exon arrangements bear the marks of gene family evolution [56, 57]. This is consistent with the results previously reported by researchers that plants tend to retain genes that do not contain introns or contain short introns during evolution [58]. The *nsLTP* family formed during the early differentiation of terrestrial plants and evolved new subclasses during terrestrial plant evolution [5, 42]. Unlike animals, genes with few or no introns in plants are thought to be expressed at lower levels [59]. However, a compact genetic structure may help genes to respond to endogenous and/or exogenous stimuli by allowing rapid expression [60].

The character of genetic duplication in the origins of evolutionary novelty, complexity and the expansion of gene families has long been recognized [56, 57, 61]. The major duplication patterns reported are tandem duplication and segmental duplication [62]. In our findings, both tandem and segmental duplication events played an important role in potato *nsLTP* amplification. *StnsLTPs* are located on ten potato chromosomes. The number of *nsLTPs* identified in potato exceeds those previously identified in *Arabidopsis*, rice, wheat, and maize. Although the genome size of potato(844 Mb) [33]is close to seven times as that of *Arabidopsis*(125 Mb) [63], the number of *nsLTP* genes in potato (83 genes) is 1.69 times the number of *nsLTP* genes in *Arabidopsis* (49 genes). The key reason for this visible fact is that the evolution of this gene family may be complex and affected by gene duplication and gene loss. The *nsLTP* gene family may have lost genes during the whole-genome duplication of the potato. A grand total of 19 duplicated *StnsLTPs* gene pairs were found, including 16 tandem duplicated gene clusters and three pairs of segmentally duplicated genes. As shown in Additional file 3: Table S3, we found that a tandem duplicated gene pair already existed before the segmental duplication event occurred in the formation of the *StnsLTP* gene family. Gene duplication is essential in the expansion and functional diversification of the gene family [64]. This is very similar to our research. Our analysis showed that duplication events not only contribute to the amplification of the *StnsLTP* genes family but also cause genetic diversity in the evolutionary process. Previous studies have shown that duplicated *nsLTP* genes have similar expression patterns.

Gene expression patterns found by RNA-seq of *nsLTP* genes in different organs and/or tissues have been reported in a large number of species, such as *Arabidopsis*, rice, peppers, and tomatoes. We used the available potato RNA-seq data [33] to analyze the expression pattern profiles of *nsLTPs* in different tissues/organs to elucidate their functional roles. From the heat map (Fig. 6), the expression levels of most *StnsLTP* genes in various tissues/organs are significantly different, which suggests that there are differences in the functions of different *StnsLTPs*. Among these genes, *StnsLTPII.2*, *StnsLTPIV.3*, *StnsLTPVIII.1*, *StnsLTPVIII.9*, *StnsLTPVIII.11*, and *StnsLTPXIII.8* were highly expressed in the storage organs of both DM and RH (Fig. 6). Compared with the results of RNA-seq, the results of qRT-PCR showed that *StnsLTPII.2, StnsLTPVIII.9, and StnsLTPXIII.8* were highly expressed in stolons and/or young tubers (Fig. 7), indicating that these genes may be involved in tuberization and/or tuber development. Many studies have shown that nsLTPs are involved in sexual reproduction processes such as pollen development, pollen wall formation and fertilization [65, 66]. We found that *StnsLTPI.2, StnsLTPI.3, StnsLTPI.4*, *StnsLTPI.5*, *StnsLTPI.29*, *StnsLTPI.36*, *StnsLTPIV.10*, and *StnsLTPXIII.3* are highly expressed in flowers, indicating that these genes may be involved in flower development process. This is very similar to the results of qRT-PCR. However, the expression profiles generated by qRT-PCR are not always consistent with the expression pattern represented by the RNA-seq data. For example, for *StnsLTPVIII.1*, in the RNA-seq data, the part with the highest expression is the floral organ, but the result of qRT-PCR result shows the stolon. There may be many reasons for such discrepancies. The potato RNA-seq data used in our study are expressed as FPKM values. FPKM values may be affected by highly expressed genes [67]. Therefore, the deviation of the FPKM value results in a difference from the result of qRT-PCR. For the potato, which is the fourth largest food crop in the world, we are most concerned about the development of tubers. The tubers of potato are metamorphic organs formed by swelling of the tops of the underground stolons. Due to the rapid increase in starch content at the top of the stolon and the sharp reduction in reducing sugar content at the initial stage of tuber formation, Hawker et al. suggested that starch synthesis may be necessary for tuber development [68]. It is now generally accepted that the formation of tubers is caused by an increase in the number and size of cells in outer medulla, cortex, and inner medulla [69]. In previous studies, xylogen-like arabinogalactan protein was found to be an arabinogalactan protein with an nsLTP domain that can boost xylem cell formation [70]. In a previous study, it was found that the expression of some members of the *StnsLTP* family is highly tissue-specific and occurs at a critical stage in the life cycle of potato tubers [71]. We also found that some genes are highly expressed in potato tubers, such as *StnsLTPXIII.8*. Therefore, they may be xylogen-arabinogalactan proteins that are involved in xylem formation in potato tubers.

The pattern of gene expression is also affected by the promoter region, which contains transcription factor binding sites. The number and type of cis-acting elements involved in a promoter are very important for gene expression under various stresses, including light, heat, drought, and wounding, and hormone signaling (e.g., ethylene, gibberellin, cytokinin, auxin). Plant hormones are trace organic substances that are synthesized in plants and have significant effects on plant growth. Therefore, understanding the type and number of cis-acting elements related to hormones involved in the promoter is of great significance for the future studies of the *StnsLTP* gene family in regulating the molecular mechanisms of potato tuber development. Among the many hormones associated with plant growth, GA3 and ABA are recognized as the most relevant factors for tuber formation. GA3 inhibits tuber formation, while ABA promotes tuber formation [72]. Moreover, several studies have shown that *nsLTP* genes can respond to certain signaling molecules, such as abscisic acid, salicylic acid and ethylene [73–76]. In addition, we found that the promoters of the *StnsLTP* gene family contain a wide variety of cis-acting elements associated with hormonal responses, such as the GARE motif and CGTCA motif. This indicates that some members of the *StnsLTP* gene family respond to hormonal signaling molecules during potato growth and development. For example, *StnsLTPI.29* contains the most cis-acting elements that respond to hormones. Previous studies have shown that different *nsLTP* gene expression can produce different responses to abiotic stress [77]. The expression of *nsLTP* genes is usually induced by environmental factors such as low temperature [73, 74, 78, 79], drought [74, 80], and high temperature [74, 80]. In *Arabidopsis*, *AtLTPg4* plays an important role in wax and/or keratin monomer transport [81]. *AtLTP2* is involved in the integrity of the cell wall of the stratum corneum [22], and *AtLTP3* responds to freezing and drought stress signals [24]. To date, the most in-depth study of nsLTPs in potato has examined the StnsLTP.1 protein [29], which can respond to a variety of abiotic stresses, including the main environmental factors that limit potato production - heat, drought, and salinity [34]. In addition, we analyzed its cis-acting elements and discovered that they include not only cis-acting elements that respond to abiotic stress, such as HSE, and TC-rich repeats but also other functional cis-acting elements, such as ABRE, that respond to abscisic acid.

## Conclusion

The *nsLTP* family is found in a variety of plant species and participates in a variety of developmental and physiological processes. However, there has been a lack of information about this family in the potato genome. In this study, we performed a genome-wide analysis of the *nsLTP* family in potato and identified 83 *StnsLTP* genes. This study predicted and analyzed the different biochemical characteristics of StnsLTP proteins. Based on sequence similarity in the ECM region, StnsLTP proteins are divided into 8 types (I, II, IV, V, VII, VIII, XII, and XIII). Subsequently, a variety of bioinformatics methods and qRT-PCR methods were used to analyze gene structure, phylogeny, chromosomal location, gene duplication, and KaKs ratios, cis-acting elements associated with growth and development, GO annotation and expression patterns in different tissues. Furthermore, the *StnsLTP* genes in potato identified by us provide not only the basis for functional research into the *StnsLTP* genes but also a valuable reference for further study of the biological functions of the gene family and its related pathways and mechanisms.

## Additional files

Additional file 1: **Table S1.** Primers used for qRT-PCR. (DOCX 14 kb)

Additional file 2: **Table S2.**nsLTP genes identified in the potato genome and features of the deduced proteins. (DOCX 30 kb)

Additional file 3: **Table S3.**Ka/Ks analysis for the duplicated gene pairs. (DOCX 18 kb)

Additional file 4: **Table S4.** Gene names in each category of GO annotation. (DOCX 13 kb)

Additional file 5: **Figure S1.** Multiple sequence alignment of StnsLTPs. The α motif was marked with “α 1–5”. The lowest number represents cysteine residues. (TIF 16297 kb)

Additional file 6: **Figure S2.** Cis-acting elements in the *StnsLTPs’* promoter was predicted. The promoter sequences of 83 *StnsLTP* genes were analyzed by PlantCARE (region from the transcription start site to the upstream 1500 bp region). A, Elements related to phytohormone response. B, Elements related to biotic and abiotic stress. C, Elements related to regulating plant growth and development. (TIF 21401 kb)

## Abbreviations

AA: Amino acid
BLASTp: Basic local alignment search tool-protein
HMM: Hidden markov model
ORF: Open reading frame
PGDD: Plant genome duplication database
PGSC: Potato genome sequencing consortium
QRT-PCR: Quantitative real-time polymerase chain reaction
tBLASTn: Protein to translated nucleotide BLAST
