# Supplementary material for: Genome-wide identification, characterization and expression analysis of the non-specific lipid transfer proteins in potato
Source: BMC Genomics. 2019 May 14;20:375. doi: 10.1186/s12864-019-5698-x (PMC6518685; doi:10.1186/s12864-019-5698-x)
Supplement: Supplementary file 1 — Table S1. Primers used for qRT-PCR. (DOCX 14 kb) [file 12864_2019_5698_MOESM1_ESM.docx]

| **Table S1: Primers used for qRT-PCR** | | | |
| --- | --- | --- | --- |
| **Gene** | **ID** | **Forward Primer (5'-3')** | **Reverse Primer (5'-3')** |
| *StnsLtpI.2* | PGSC0003DMG400011952 | GGTGTTGTGGTGGTGTTA | TCTTGTAAGGAATGCTGACT |
| *StnsLtpI.3* | PGSC0003DMG400011953 | GATTGCATGCTTTGTGCTTTTG | AGCCAAGGAAGATGTAACTTGA |
| *StnsLtpI.4* | PGSC0003DMG400011954 | CACGGTTCAGTAAGGTAGATCA | CAAAACTCCCAAAAACACAACG |
| *StnsLtpI.7* | PGSC0003DMG400002471 | CTGGTATCCCTAGAGTTTGTGG | TCAATCTTACTGGACCTTGGAG |
| *StnsLtpI.11* | PGSC0003DMG400025988 | AGACGCTAATATGACACCATGT | CATCTTTAAGGTTGGCGTATCG |
| *StnsLtpI.14* | PGSC0003DMG400001322 | GTTCATGTTGTGATGGTGTGAA | TAGCAGTAGTAATACCACAGCG |
| *StnsLtpI.26* | PGSC0003DMG400016102 | GGTGTATTCTAACCTCGAACCA | AGGAGAGATTTAAGTCCACTGC |
| *StnsLtpI.29* | PGSC0003DMG400001904 | CCACATCTTATTCAATGGCTGG | ACCAAGGCACGGGATTATATAG |
| *StnsLtpI.30* | PGSC0003DMG400012839 | GAGTTTGTGGAGTCAACATTCC | CTTATTCTTCATCTCCGCAAGC |
| *StnsLtpI.31* | PGSC0003DMG400012838 | GTGGCGTAAACATTCCTTACAA | TCTCCGCAAGCTATATCACATT |
| *StnsLtpI.32* | PGSC0003DMG400012837 | GGTGTTGTGGTGGTATTAAG | AATGTTTACGCCACAAACT |
| *StnsLtpI.34* | PGSC0003DMG402031237 | TGTTGTGGTGGAGTTAAGG | AATGTTTACGCCACAAACG |
| *StnsLtpI.35* | PGSC0003DMG401031237 | GGTGTTGTGGTGGAGTTAA | AATGTTTACGCCACAAACA |
| *StnsLtpI.36* | PGSC0003DMG400040954 | GACAGTGTTGTGATGTTGTT | GTGGAGAGGCTAATCTTGAA |
| *StnsLtpII.2* | PGSC0003DMG400021763 | CTCAAGAATCCAAGCCTTAAGC | AAGACCTAACATGGCTTAGGAG |
| *StnsLtpII.5* | PGSC0003DMG400005731 | CTCATGAGAGAGTCACTGTGTG | GCCAATGATGCATGATAGAGAC |
| *StnsLtpV.2* | PGSC0003DMG400028909 | GGATGAGAATGCATCTGTTTCC | TAACAGCACAAAGACATTTCGG |
| *StnsLtpVIII.1* | PGSC0003DMG400013434 | CCAATGATGCAAGTTTACCCAA | CAAAGACACATTTTGCAGAGGA |
| *StnsLtpVIII.9* | PGSC0003DMG400004782 | TAGGGAATTGACAGATTGCACT | AAAGAATGGAAGCTCTTGGGTA |
| *StnsLtpXIII.5* | PGSC0003DMG400034309 | AGTCCTCAGTTGAAGAGTATG | CGGTGATGATGATGATGATG |
| *StnsLtpXIII.7* | PGSC0003DMG400020131 | GGTGGTGCTTCTTCTTCTT | GATGGTGGTGAATGATGCT |
| *StnsLtpXIII.8* | PGSC0003DMG400022295 | TTGCAAGGTCACAATCAATGAG | CCAAATTTTCCCAACTCGGATT |
