# Supplementary material for: Genome-wide identification, characterization and expression analysis of the non-specific lipid transfer proteins in potato
Source: BMC Genomics. 2019 May 14;20:375. doi: 10.1186/s12864-019-5698-x (PMC6518685; doi:10.1186/s12864-019-5698-x)
Supplement: Supplementary file 2 — Table S2.nsLTP genes identified in the potato genome and features of the deduced proteins. (DOCX 30 kb) [file 12864_2019_5698_MOESM2_ESM.docx]

| **Table S2: The detailed information of potato nsLTP gene family** | | | | | | | | |
| --- | --- | --- | --- | --- | --- | --- | --- | --- |
| **Gene**^a^ | **Locus ID^c^** | **Chromosome location^d^** | **CDS^e^ length (bp)** | **Introns^f^** | **Signal peptide^g^** | **Protein length (aa)^h^** | **Mw (kD)^h^** | **pI^h^** |
| **Type I** |  |  |  |  |  |  |  |  |
| *StnsLTPI.1*^ij^ | PGSC0003DMG400011951 | ch00:22013874..22014389 | 345 | 1 | Y | 114 | 11.38 | 8.34 |
| *StnsLTPI.2*^j^ | PGSC0003DMG400011952 | ch00:22036158..22036674 | 345 | 1 | Y | 114 | 11.36 | 8.34 |
| *StnsLTPI.3* ^j^ | PGSC0003DMG400011953 | ch00:22057167..22057845 | 339 | 1 | Y | 112 | 11.39 | 8.58 |
| *StnsLTPI.4* ^j^ | PGSC0003DMG400011954 | ch00:22081389..22082057 | 345 | 1 | Y | 114 | 11.47 | 8.73 |
| *StnsLTPI.5* ^j^ | PGSC0003DMG400011955 | ch00:22099198..22100312 | 342 | 1 | Y | 113 | 11.8 | 9.11 |
| *StnsLTPI.6* ^j^ | PGSC0003DMG400011949 | ch00:22111758..22112075 | 318 | 0 | N | 105 | 11.25 | 9.30 |
| *StnsLTPI.7* ^j^ | PGSC0003DMG400002471 | ch01:50944551..50945051 | 345 | 1 | Y | 114 | 11.47 | 9.12 |
| *StnsLTPI.8* ^j^ | PGSC0003DMG400031127 | ch01:67139309..67140324 | 366 | 1 | Y | 121 | 12.39 | 8.62 |
| *StnsLTPI.9* | PGSC0003DMG400031126 | ch01:67141270..67142003 | 363 | 1 | Y | 120 | 13.05 | 9.20 |
| *StnsLTPI.10* | PGSC0003DMG400031125 | ch01:67142780..67143270 | 414 | 1 | Y | 137 | 14.85 | 8.94 |
| *StnsLTPI.11* | PGSC0003DMG400025988 | ch01:69558764..69559120 | 357 | 0 | Y | 118 | 12.14 | 8.14 |
| *StnsLTPI.12* | PGSC0003DMG400025987 | ch01:69566405..69566947 | 348 | 1 | Y | 115 | 11.74 | 8.47 |
| *StnsLTPI.13* | PGSC0003DMG400000218 | ch01:73368610..73368948 | 339 | 0 | Y | 112 | 12.37 | 9.53 |
| *StnsLTPI.14* | PGSC0003DMG400001322 | ch02:46262505..46262930 | 336 | 1 | Y | 111 | 11.36 | 7.47 |
| *StnsLTPI.15* | PGSC0003DMG400020823 | ch06:4194079..4194432 | 354 | 0 | Y | 117 | 12.68 | 9.48 |
| *StnsLTPI.16* | PGSC0003DMG400020837 | ch06:4212719..4213075 | 357 | 0 | Y | 118 | 12.89 | 9.11 |
| *StnsLTPI.17* | PGSC0003DMG400020822 | ch06:4222288..4222641 | 354 | 0 | Y | 117 | 12.75 | 8.6 |
| *StnsLTPI.18* | PGSC0003DMG400020821 | ch06:4230352..4230711 | 360 | 0 | Y | 119 | 12.68 | 8.08 |
| *StnsLTPI.19* | PGSC0003DMG400044985 | ch06:46252398..46252706 | 309 | 0 | N | 102 | 10.45 | 4.83 |
| *StnsLTPI.20* | PGSC0003DMG400041359 | ch06:46253617..46253991 | 375 | 0 | Y | 124 | 12.62 | 8.79 |
| *StnsLTPI.21* | PGSC0003DMG400025537 | ch08:41101826..41102197 | 372 | 0 | Y | 123 | 12.7 | 8.55 |
| *StnsLTPI.22* | PGSC0003DMG400025538 | ch08:41110151..41110519 | 369 | 0 | Y | 122 | 12.74 | 9.46 |
| *StnsLTPI.23* | PGSC0003DMG400025539 | ch08:41113026..41113403 | 378 | 0 | Y | 125 | 13.16 | 7.45 |
| *StnsLTPI.24* | PGSC0003DMG400044717 | ch08:41117522..41117842 | 321 | 0 | N | 106 | 11.02 | 9.34 |
| *StnsLTPI.25* | PGSC0003DMG400016103 | ch08:41195063..41195428 | 366 | 0 | Y | 121 | 13.05 | 9.34 |
| *StnsLTPI.26* ^j^ | PGSC0003DMG400016102 | ch08:41198048..41198398 | 351 | 0 | Y | 116 | 11.81 | 8.42 |
| *StnsLTPI.27* | PGSC0003DMG400016105 | ch08:41208805..41209176 | 372 | 0 | Y | 123 | 13.56 | 9.70 |
| *StnsLTPI.28* | PGSC0003DMG400015092 | ch08:41264878..41265195 | 318 | 0 | N | 105 | 10.78 | 8.87 |
| *StnsLTPI.29* ^j^ | PGSC0003DMG400001904 | ch09:16527725..16528906 | 333 | 1 | Y | 110 | 11.11 | 9.00 |
| *StnsLTPI.30*^k j^ | PGSC0003DMG400012839 | ch10:13444868..13445387 | 345 | 1 | Y | 114 | 11.41 | 8.34 |
| *StnsLTPI.31* ^j^ | PGSC0003DMG400012838 | ch10:13478824..13479362 | 345 | 1 | Y | 114 | 11.36 | 8.87 |
| *StnsLTPI.32* | PGSC0003DMG400012837 | ch10:13483715..13484124 | 357 | 1 | Y | 118 | 11.89 | 9.21 |
| *StnsLTPI.33* | PGSC0003DMG400031236 | ch10:48904555..48905098 | 342 | 1 | Y | 113 | 11.52 | 8.58 |
| *StnsLTPI.34* ^j^ | PGSC0003DMG402031237 | ch10:48915371..48915727 | 357 | 0 | Y | 118 | 11.92 | 8.85 |
| *StnsLTPI.35* | PGSC0003DMG401031237 | ch10:48918720..48919070 | 351 | 0 | Y | 116 | 11.61 | 8.98 |
| *StnsLTPI.36* | PGSC0003DMG400040954 | ch10:49170543..49171657 | 342 | 1 | Y | 113 | 11.8 | 9.11 |
| **Type II** |  |  |  |  |  |  |  |  |
| *StnsLTPII.1* | PGSC0003DMG400026381 | ch02:41505128..41505412 | 285 | 0 | Y | 94 | 9.77 | 8.37 |
| *StnsLTPII.2* | PGSC0003DMG400021763 | ch03:5152184..5152462 | 276 | 0 | Y | 92 | 9.95 | 8.88 |
| *StnsLTPII.3* | PGSC0003DMG400021761 | ch03:5190417..5190707 | 291 | 0 | Y | 96 | 10.65 | 7.48 |
| *StnsLTPII.4* | PGSC0003DMG400017133 | ch03:5280386..5280676 | 291 | 0 | Y | 96 | 10.44 | 8.45 |
| *StnsLTPII.5* ^j^ | PGSC0003DMG400005731 | ch03:58758002..58758283 | 282 | 0 | Y | 93 | 9.88 | 9.44 |
| *StnsLTPII.6* | PGSC0003DMG400030587 | ch05:3454127..3454414 | 288 | 0 | Y | 95 | 10.42 | 9.41 |
| **Type IV** |  |  |  |  |  |  |  |  |
| *StnsLTPIV.1* | PGSC0003DMG400032487 | ch00:41242022..41242438 | 417 | 0 | Y | 138 | 14.64 | 9.76 |
| *StnsLTPIV.2* | PGSC0003DMG400011323 | ch01:58072193..58072504 | 312 | 0 | Y | 103 | 10.77 | 7.48 |
| *StnsLTPIV.3*^j^ | PGSC0003DMG400012567 | ch01:81769725..81772029 | 558 | 2 | Y | 185 | 18.68 | 5.24 |
| *StnsLTPIV.4* ^j^ | PGSC0003DMG400025168 | ch01:85660585..85660899 | 315 | 0 | Y | 104 | 10.95 | 7.47 |
| *StnsLTPIV.5* | PGSC0003DMG400005630 | ch03:59098800..59099679 | 558 | 2 | Y | 185 | 19.54 | 5.05 |
| *StnsLTPIV.6* | PGSC0003DMG400002487 | ch03:61229623..61229913 | 291 | 0 | Y | 96 | 10.08 | 7.51 |
| *StnsLTPIV.7* | PGSC0003DMG400032819 | ch07:49443508..49443982 | 378 | 1 | Y | 125 | 14.43 | 6.49 |
| *StnsLTPIV.8* | PGSC0003DMG400032260 | ch09:53974975..53977208 | 666 | 3 | Y | 221 | 22.88 | 5.78 |
| *StnsLTPIV.9* | PGSC0003DMG400044023 | ch10:6552952..6553299 | 348 | 0 | Y | 115 | 12.38 | 8.65 |
| *StnsLTPIV.10* | PGSC0003DMG400027400 | ch11:43618340..43620996 | 621 | 3 | Y | 206 | 21.29 | 4.49 |
| **Type V** |  |  |  |  |  |  |  |  |
| *StnsLTPV.1* | PGSC0003DMG400021691 | ch03:33400297..33400723 | 339 | 1 | Y | 112 | 11.68 | 8.08 |
| *StnsLTPV.2* | PGSC0003DMG400028909 | ch06:37838693..37839117 | 342 | 1 | Y | 113 | 11.85 | 8.79 |
| **Type VII** |  |  |  |  |  |  |  |  |
| *StnsLTPVII.1* | PGSC0003DMG400020710 | ch01:79840175..79840657 | 369 | 1 | N | 122 | 12.94 | 8.12 |
| **Type VIII** |  |  |  |  |  |  |  |  |
| *StnsLTPVIII.1* | PGSC0003DMG400013434 | ch03:296862..297944 | 507 | 2 | Y | 168 | 17.15 | 8.71 |
| *StnsLTPVIII.2* | PGSC0003DMG400031724 | ch03:58157196..58157597 | 402 | 0 | Y | 133 | 14.17 | 8.04 |
| *StnsLTPVIII.3* | PGSC0003DMG400005678 | ch03:58196812..58198326 | 537 | 2 | Y | 178 | 18.24 | 7.51 |
| *StnsLTPVIII.4* | PGSC0003DMG402016309 | ch06:40329841..40330554 | 450 | 1 | Y | 149 | 15.07 | 8.63 |
| *StnsLTPVIII.5* | PGSC0003DMG401016309 | ch06:40331529..40332059 | 444 | 1 | Y | 147 | 15.59 | 4.43 |
| *StnsLTPVIII.6* | PGSC0003DMG402020132 | ch06:58973492..58974574 | 555 | 2 | Y | 184 | 18.17 | 8.16 |
| *StnsLTPVIII.7* | PGSC0003DMG400012481 | ch08:440236..442510 | 420 | 2 | N | 139 | 14.08 | 4.36 |
| *StnsLTPVIII.8* | PGSC0003DMG400043645 | ch08:52837306..52837823 | 360 | 1 | Y | 119 | 12.36 | 5.17 |
| *StnsLTPVIII.9* | PGSC0003DMG400004782 | ch08:52840739..52843635 | 456 | 2 | Y | 151 | 15.64 | 8.12 |
| *StnsLTPVIII.10* | PGSC0003DMG400019323 | ch09:47954002..47955133 | 558 | 2 | Y | 185 | 18.33 | 4.66 |
| *StnsLTPVIII.11* | PGSC0003DMG400032250 | ch09:53961326..53962627 | 495 | 2 | Y | 164 | 16.8 | 4.39 |
| *StnsLTPVIII.12* | PGSC0003DMG400032249 | ch09:53969664..53971648 | 525 | 2 | Y | 174 | 17.23 | 8.41 |
| **Type XII** |  |  |  |  |  |  |  |  |
| *StnsLTPXII.1* | PGSC0003DMG400030768 | ch06:41296678..41299812 | 396 | 1 | N | 131 | 14.22 | 4.17 |
| *StnsLTPXII.2* | PGSC0003DMG400043006 | ch06:41308060..41308416 | 357 | 0 | Y | 118 | 12.72 | 4.16 |
| *StnsLTPXII.3* | PGSC0003DMG400041307 | ch10:6496379..6496717 | 339 | 0 | Y | 112 | 12.22 | 7.5 |
| *StnsLTPXII.4* | PGSC0003DMG400035278 | ch10:6508566..6508904 | 339 | 0 | Y | 112 | 11.9 | 8.44 |
| *StnsLTPXII.5* | PGSC0003DMG400036647 | ch10:6525230..6525568 | 339 | 0 | Y | 112 | 11.99 | 8.99 |
| *StnsLTPXII.6* | PGSC0003DMG400038800 | ch10:6533580..6533927 | 348 | 0 | Y | 115 | 12.55 | 6.69 |
| *StnsLTPXII.7* | PGSC0003DMG400045247 | ch11:15727573..15727920 | 348 | 0 | Y | 115 | 12.69 | 8.44 |
| **Type XIII** |  |  |  |  |  |  |  |  |
| *StnsLTPXIII.1* | PGSC0003DMG400032182 | ch01:1516696..1518117 | 573 | 2 | Y | 190 | 20.26 | 7.51 |
| *StnsLTPXIII.2* | PGSC0003DMG400028977 | ch01:67049409..67050818 | 582 | 2 | Y | 193 | 20.31 | 7.99 |
| *StnsLTPXIII.3* | PGSC0003DMG400021773 | ch03:5197954..5198256 | 303 | 0 | Y | 100 | 10.95 | 8.35 |
| *StnsLTPXIII.4* | PGSC0003DMG400014320 | ch03:42442293..42442631 | 339 | 0 | Y | 112 | 11.66 | 4.16 |
| *StnsLTPXIII.5* | PGSC0003DMG400034309 | ch05:14008409..14012146 | 594 | 1 | Y | 197 | 20.43 | 5.63 |
| *StnsLTPXIII.6* | PGSC0003DMG400028778 | ch06:50802779..50803376 | 495 | 1 | N | 164 | 17.01 | 5.07 |
| *StnsLTPXIII.7* | PGSC0003DMG400020131 | ch06:58976338..58976986 | 456 | 2 | Y | 151 | 15.51 | 8.40 |
| *StnsLTPXIII.8* | PGSC0003DMG400022295 | ch07:55244690..55244998 | 309 | 0 | Y | 102 | 10.89 | 9.28 |
| *StnsLTPXIII.9* | PGSC0003DMG400019325 | ch09:47944270..47944611 | 342 | 0 | Y | 113 | 12.05 | 8.66 |

^a^ Systematic designation given to potato nsLtp genes

^b^ Genes identified and named by their predecessors

^c^ Locus identity number of nsLtp assigned by PGSC (Potato Genome Sequencing Consortium, http://solanaceae.plantbiology.msu.edu/integrated_searches.shtml)

^d^ Chromosomal localization of potato nsLtp genes.

^e^ Length of open Coding sequence (CDS). bp, base pair.

^f^ Number of introns obtained from PGSC (Potato Genome Sequencing Consortium, http://solanaceae.plantbiology.msu.edu/integrated_searches.shtml).

^g^ N-terminal signal sequence predicted by PGSC (Potato Genome Sequencing Consortium, http://solanaceae.plantbiology.msu.edu/integrated_searches.shtml).Y,Yes;N,No.

^h^ Physicochemical properties of mature proteins, of which protein length (aa, amino acid), isoelectric point (pI)and molecular weight (Mw) are predicted to pass the Expasy website (http://web.expasy.org/protparam/).

^i^ This gene was identified by Gangadhar et al. (2016) and named *StnsLTP1*.

^j^ These genes were identified and named by Liu et al(2010).

^k^ This gene was identified by Gao et al. (2009) and named *StLTPa7*.
