# Supplementary material for: Genome-wide identification, characterization and expression analysis of the non-specific lipid transfer proteins in potato
Source: BMC Genomics. 2019 May 14;20:375. doi: 10.1186/s12864-019-5698-x (PMC6518685; doi:10.1186/s12864-019-5698-x)
Supplement: Supplementary file 3 — Table S3.Ka/Ks analysis for the duplicated gene pairs. (DOCX 18 kb) [file 12864_2019_5698_MOESM3_ESM.docx]

| **Table S3: Ka/Ks analysis for the duplicated gene pairs** | | | | | | | |
| --- | --- | --- | --- | --- | --- | --- | --- |
| **Duplicated gene 1** | **Duplicated gene 2** | **Ka** | **Ks** | **Ka/Ks** | **Purifng selection** | **Duplicate type** | **Age (MYA)** |
| *StnsLTPI.8* | *StnsLTPI.9* | 0.767702 | 1.71366 | 0.44799 | YES | tandem | 329.55 |
| *StnsLTPI.9* | *StnsLTPI.10* | 0.723009 | 3.26708 | 0.221301 | YES | tandem | 628.28 |
| *StnsLTPI.8* | *StnsLTPI.10* | 0.872113 | 3.29826 | 0.264416 | YES | tandem | 634.28 |
| *StnsLTPI.11* | *StnsLTPI.12* | 0.112335 | 0.336995 | 0.333342 | YES | tandem | 64.81 |
| *StnsLTPII.2* | *StnsLTPII.3* | 0.534639 | 0.686354 | 0.778954 | YES | tandem | 131.99 |
| *StnsLTPII.3* | *StnsLTPXIII.3* | 0.491975 | 0.911338 | 0.539838 | YES | tandem | 175.26 |
| *StnsLTPXIII.3* | *StnsLTPII.2* | 0.344816 | 0.654908 | 0.526511 | YES | tandem | 125.94 |
| *StnsLTPVIII.2* | *StnsLTPVIII.3* | 0.0317035 | 0.0584264 | 0.542623 | YES | tandem | 11.24 |
| *StnsLTPVIII.1* | *StnsLTPVIII.5* | 0.31209 | 0.620485 | 0.502977 | YES | segmental | 119.32 |
| *StnsLTPI.15* | *StnsLTPI.16* | 0.33578 | 0.632679 | 0.530727 | YES | tandem | 121.67 |
| *StnsLTPI.16* | *StnsLTPI.17* | 0.333865 | 0.566352 | 0.589501 | YES | tandem | 108.91 |
| *StnsLTPI.17* | *StnsLTPI.18* | 0.477733 | 1.43311 | 0.333353 | YES | tandem | 275.60 |
| *StnsLTPI.18* | *StnsLTPI.15* | 0.311475 | 0.931162 | 0.334501 | YES | tandem | 179.07 |
| *StnsLTPI.16* | *StnsLTPI.18* | 0.383203 | 0.729742 | 0.525122 | YES | tandem | 140.34 |
| *StnsLTPI.17* | *StnsLTPI.15* | 0.139256 | 0.330814 | 0.42095 | YES | tandem | 63.62 |
| *StnsLTPVIII.5* | *StnsLTPVIII.4* | 0.631107 | 3.09268 | 0.204065 | YES | tandem | 594.75 |
| *StnsLTPVIII.5* | *StnsLTPVIII.11* | 0.265717 | 0.910267 | 0.291911 | YES | segmental | 175.05 |
| *StnsLTPXII.1* | *StnsLTPXII.2* | 0.388939 | 0.43135 | 0.901679 | YES | tandem | 82.95 |
| *StnsLTPI.19* | *StnsLTPI.20* | 0.352681 | 1.63614 | 0.215557 | YES | tandem | 314.64 |
| *StnsLTPVIII.6* | *StnsLTPXIII.7* | 0.517209 | 3.35889 | 0.153982 | YES | tandem | 645.94 |
| *StnsLTPI.21* | *StnsLTPI.22* | 0.345853 | 1.67477 | 0.206508 | YES | tandem | 322.07 |
| *StnsLTPI.22* | *StnsLTPI.23* | 0.563935 | 1.7848 | 0.315966 | YES | tandem | 343.23 |
| *StnsLTPI.23* | *StnsLTPI.24* | 0.228244 | 0.318943 | 0.715627 | YES | tandem | 61.34 |
| *StnsLTPI.24* | *StnsLTPI.21* | 0.620287 | 4.92384 | 0.125976 | YES | tandem | 946.89 |
| *StnsLTPI.22* | *StnsLTPI.24* | 0.568718 | 1.37313 | 0.414176 | YES | tandem | 264.06 |
| *StnsLTPI.21* | *StnsLTPI.23* | 0.641146 | 1.89935 | 0.337561 | YES | tandem | 365.26 |
| *StnsLTPI.25* | *StnsLTPI.26* | 0.268445 | 0.641443 | 0.418502 | YES | tandem | 123.35 |
| *StnsLTPI.26* | *StnsLTPI.27* | 0.361976 | 0.60853 | 0.594837 | YES | tandem | 117.03 |
| *StnsLTPI.27* | *StnsLTPI.25* | 0.276374 | 0.404323 | 0.683548 | YES | tandem | 77.75 |
| *StnsLTPVIII.8* | *StnsLTPVIII.9* | 0.164603 | 0.256881 | 0.640773 | YES | tandem | 49.40 |
| *StnsLTPXIII.7* | *StnsLTPXIII.9* | 0.235646 | 2.21579 | 0.106348 | YES | segmental | 426.11 |
| *StnsLTPVIII.10* | *StnsLTPXIII.9* | 0.502346 | 3.13755 | 0.160108 | YES | tandem | 603.38 |
| *StnsLTPVIII.11* | *StnsLTPVIII.12* | 0.563028 | 3.51005 | 0.160404 | YES | tandem | 675.01 |
| *StnsLTPVIII.12* | *StnsLTPIV.8* | 0.827416 | 2.72443 | 0.303702 | YES | tandem | 523.93 |
| *StnsLTPIV.8* | *StnsLTPVIII.11* | 0.609324 | 3.49918 | 0.174133 | YES | tandem | 672.92 |
| *StnsLTPXII.3* | *StnsLTPXII.4* | 0.131181 | 0.111812 | 1.17323 | NO | tandem | 21.50 |
| *StnsLTPXII.4* | *StnsLTPXII.5* | 0.110767 | 0.141625 | 0.782111 | YES | tandem | 27.24 |
| *StnsLTPXII.5* | *StnsLTPXII.6* | 0.182399 | 0.167592 | 1.08835 | NO | tandem | 32.23 |
| *StnsLTPXII.6* | *StnsLTPXII.3* | 0.267616 | 0.260463 | 1.02746 | NO | tandem | 50.09 |
| *StnsLTPXII.3* | *StnsLTPXII.5* | 0.194516 | 0.435325 | 0.44683 | YES | tandem | 83.72 |
| *StnsLTPXII.4* | *StnsLTPXII.6* | 0.255041 | 0.032513 | 7.84429 | NO | tandem | 6.25 |
| *StnsLTPI.30* | *StnsLTPI.31* | 0.0735788 | 0.389273 | 0.189016 | YES | tandem | 74.86 |
| *StnsLTPI.31* | *StnsLTPI.32* | 0.0562763 | 0.202994 | 0.277231 | YES | tandem | 39.04 |
| *StnsLTPI.32* | *StnsLTPI.30* | 0.0569444 | 0.492921 | 0.115524 | YES | tandem | 94.79 |
| *StnsLTPI.33* | *StnsLTPI.34* | 0.152369 | 0.347753 | 0.438153 | YES | tandem | 66.88 |
| *StnsLTPI.34* | *StnsLTPI.35* | 0.0122577 | 0.0204132 | 0.60048 | YES | tandem | 3.93 |
| *StnsLTPI.35* | *StnsLTPI.33* | 0.145148 | 0.321237 | 0.45184 | YES | tandem | 61.78 |
