# Supplementary material for: Genome-wide identification, characterization and expression analysis of the non-specific lipid transfer proteins in potato
Source: BMC Genomics. 2019 May 14;20:375. doi: 10.1186/s12864-019-5698-x (PMC6518685; doi:10.1186/s12864-019-5698-x)
Supplement: Supplementary file 4 — Table S4. Gene names in each category of GO annotation. (DOCX 13 kb) [file 12864_2019_5698_MOESM4_ESM.docx]

| **Table S4: Gene names in each category of GO annotation** | | |
| --- | --- | --- |
| **Molecular function** | **Number** | **Gene name** |
| lipid binding | 47 | StnsLtpI.5, StnsLtpI.6, StnsLtpI.3, StnsLtpI.4, StnsLtpI.1, StnsLtpI.2, StnsLtpI.22, StnsLtpI.9, StnsLtpI.21, StnsLtpI.20, StnsLtpI.7, StnsLtpI.8, StnsLtpI.26, StnsLtpVII.1, StnsLtpI.25, StnsLtpI.29, StnsLtpI.28, StnsLtpI.27, StnsLtpIV.3, StnsLtpIV.5, StnsLtpVIII.10, StnsLtpVIII.11, StnsLtpVIII.12, StnsLtpVIII.6, StnsLtpVIII.4, StnsLtpVIII.1, StnsLtpI.11, StnsLtpI.33, StnsLtpXII.7, StnsLtpI.10, StnsLtpI.32, StnsLtpI.31, StnsLtpXIII.2, StnsLtpI.30, StnsLtpXIII.1, StnsLtpXIII.4, StnsLtpI.14, StnsLtpI.36, StnsLtpI.13, StnsLtpI.35, StnsLtpI.12, StnsLtpI.34, StnsLtpI.19, StnsLtpI.18, StnsLtpXIII.7, StnsLtpI.16, StnsLtpXIII.9 |
| kinase activity | 1 | StnsLtpIV.9 |
|  |  |  |
| **Biological process** | **Number** | **Gene name** |
| lipid transport | 60 | StnsLtpI.22, StnsLtpI.21, StnsLtpI.20, StnsLtpI.26, StnsLtpI.25, StnsLtpI.24, StnsLtpI.23, StnsLtpI.29, StnsLtpI.28, StnsLtpI.27, StnsLtpIV.4, StnsLtpIV.6, StnsLtpVIII.10, StnsLtpVIII.11, StnsLtpVIII.12, StnsLtpVIII.6, StnsLtpVIII.4, StnsLtpVIII.1, StnsLtpXII.3, StnsLtpI.33, StnsLtpXII.7, StnsLtpI.32, StnsLtpXII.6, StnsLtpI.31, StnsLtpXII.5, StnsLtpI.30, StnsLtpXII.4, StnsLtpI.36, StnsLtpI.35, StnsLtpI.34, StnsLtpIV.10, StnsLtpI.5, StnsLtpI.6, StnsLtpI.3, StnsLtpI.4, StnsLtpI.1, StnsLtpI.2, StnsLtpI.9, StnsLtpI.7, StnsLtpI.8, StnsLtpVII.1, StnsLtpII.1, StnsLtpII.2, StnsLtpI.11, StnsLtpI.10, StnsLtpXIII.2, StnsLtpXIII.1, StnsLtpI.15, StnsLtpXIII.4, StnsLtpI.14, StnsLtpI.13, StnsLtpI.12, StnsLtpI.19, StnsLtpI.18, StnsLtpII.5, StnsLtpXIII.7, StnsLtpI.17, StnsLtpII.6, StnsLtpI.16, StnsLtpXIII.9 |
| response to biotic stimulus | 1 | StnsLtpI.29 |
| defense response | 1 | StnsLtpI.29 |
| phosphorylation | 1 | StnsLtpIV.9 |
|  |  |  |
| **Cellular component** | **Number** | **Gene name** |
| membrane | 35 | StnsLtpIV.10, StnsLtpI.5, StnsLtpI.6, StnsLtpI.3, StnsLtpI.4, StnsLtpI.22, StnsLtpI.9, StnsLtpI.21, StnsLtpI.20, StnsLtpI.8, StnsLtpVIII.9, StnsLtpI.26, StnsLtpI.25, StnsLtpI.24, StnsLtpI.23, StnsLtpI.29, StnsLtpI.28, StnsLtpI.27, StnsLtpVIII.8, StnsLtpXII.3, StnsLtpI.11, StnsLtpXII.6, StnsLtpXII.5, StnsLtpXII.4, StnsLtpI.15, StnsLtpI.36, StnsLtpI.13, StnsLtpI.35, StnsLtpXIII.6, StnsLtpI.12, StnsLtpI.34, StnsLtpI.19, StnsLtpI.18, StnsLtpI.17, StnsLtpI.16 |
| integral component of membrane | 18 | StnsLtpVIII.10, StnsLtpVIII.11, StnsLtpIV.1, StnsLtpVIII.12, StnsLtpIV.2, StnsLtpVIII.7, StnsLtpVIII.6, StnsLtpVIII.5, StnsLtpVIII.4, StnsLtpIV.8, StnsLtpIV.9, StnsLtpVIII.1, StnsLtpXIII.2, StnsLtpXIII.1, StnsLtpXIII.4, StnsLtpXIII.5, StnsLtpXIII.7, StnsLtpXIII.9 |
| anchored component of membrane | 2 | StnsLtpVIII.3, StnsLtpVIII.2 |
