# Supplementary figures and images for: Genome-wide identification, characterization and expression analysis of the non-specific lipid transfer proteins in potato
Source: BMC Genomics. 2019 May 14;20:375. doi: 10.1186/s12864-019-5698-x (PMC6518685; doi:10.1186/s12864-019-5698-x)

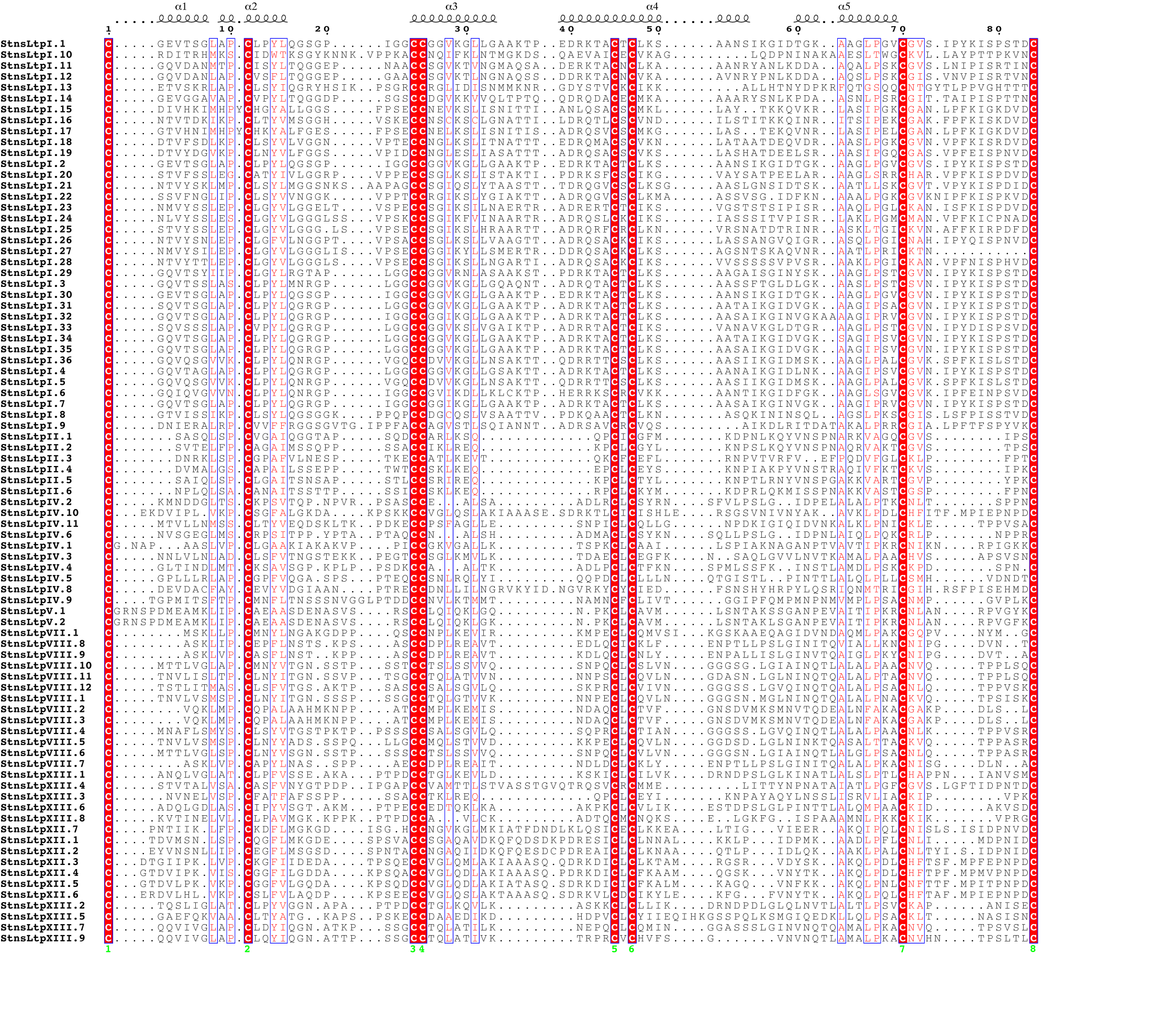

Supplement: Supplementary file 5 — Figure S1. Multiple sequence alignment of StnsLTPs. The α motif was marked with “α 1–5”. The lowest number represents cysteine residues. (TIF 16297 kb) [file 12864_2019_5698_MOESM5_ESM.tif]

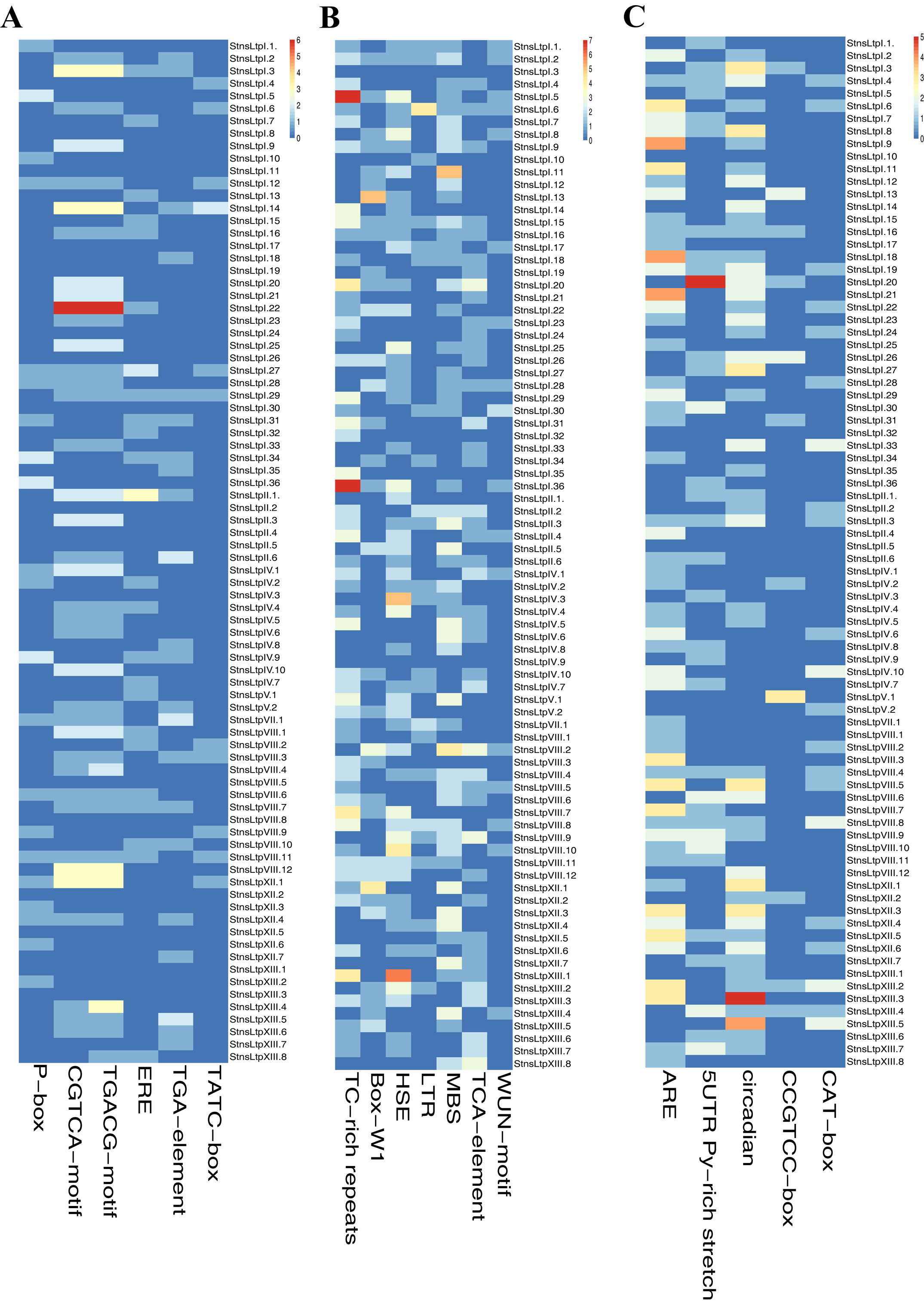

Supplement: Supplementary file 6 — Figure S2. Cis-acting elements in the StnsLTPs’ promoter was predicted. The promoter sequences of 83 StnsLTP genes were analyzed by PlantCARE (region from the transcription start site to the upstream 1500 bp region). A, Elements related to phytohormone response. B, Elements related to biotic and abiotic stress. C, Elements related to regulating plant growth and development. (TIF 21401 kb) [file 12864_2019_5698_MOESM6_ESM.tif]
